# Micro- and Macro-Anatomical Frameworks of Lymph Nodes Indispensable for the Lymphatic System Filtering Function

**DOI:** 10.3389/fcell.2022.902601

**Published:** 2022-06-20

**Authors:** Madoka Ozawa, Shihori Nakajima, Daichi Kobayashi, Koichi Tomii, Nan-Jun Li, Tomoya Watarai, Ryo Suzuki, Satoshi Watanabe, Yasuhiro Kanda, Arata Takeuchi, Tomoya Katakai

**Affiliations:** ^1^ Department of Immunology, Niigata University Graduate School of Medical and Dental Sciences, Niigata, Japan; ^2^ Department of Respiratory Medicine and Infectious Diseases, Niigata University Graduate School of Medical and Dental Sciences, Niigata, Japan; ^3^ Department of Immunology, Tokyo Medical University, Tokyo, Japan

**Keywords:** lymph fluid, lymph node chain, macrophage, medullary sinus, subcapsular sinus

## Abstract

In the lymphatic vascular system, lymph nodes (LNs) play a pivotal role in filtering and removing lymph-borne substances. The filtering function of LNs involves resident macrophages tightly associated with unique lymphatic sinus structures. Moreover, an intermittently arranged LN in the lymphatic pathway is considered to cooperatively prevent lymph-borne substances from entering blood circulation. However, the functional significance of tissue microarchitecture, cellular composition, and individual LNs in the “LN chain” system is not fully understood. To explore the mechanistic and histo-anatomical significance of LNs as lymph fluid filters, we subcutaneously injected fluorescent tracers into mice and examined the details of lymphatic transport to the LNs qualitatively and quantitatively. Lymph-borne tracers were selectively accumulated in the MARCO^+^ subcapsular-medullary sinus border (SMB) region of the LN, in which reticular lymphatic endothelial cells and CD169^+^F4/80^+^ medullary sinus macrophages construct a dense meshwork of the physical barrier, forming the main body to capture the tracers. We also demonstrated stepwise filtration *via* the LN chain in the lymphatic basin, which prevented tracer leakage into the blood. Furthermore, inflammatory responses that induce the remodeling of LN tissue as well as the lymphatic pathway reinforce the overall filtering capacity of the lymphatic basin. Taken together, specialized tissue infrastructure in the LNs and their systematic orchestration constitute an integrated filtering system for lymphatic recirculation.

## Introduction

The lymphatic system is a network of vasculature that spreads throughout the body to collect and recycle interstitial tissue fluid. The lymph fluid from peripheral tissues is returned to blood circulation through this vasculature; the initial lymphatic vessels originating in tissues gather in the collecting vessels, join the central ducts like the thoracic duct, and are finally connected to the blood at the subclavian vein (Randolph et al., 2017; Oliver et al., 2020). The unique property of the lymphatic system, which collects tissue fluid from nearly all body parts, makes it ideal and efficient for collecting foreign substances or abnormal tissue components, while it also carries the risk of spreading harmful substances throughout the body. To prevent this, lymph nodes (LNs) are positioned at key places in the lymphatic basin and function as purge filters to remove lymph-borne impure components (Lammermann and Sixt, 2008; Oliver et al., 2020). Concurrently, LNs play a central role in inducing immune responses by detecting microbial components and foreign substances in the lymph fluid (Lammermann and Sixt, 2008; Qi et al., 2014).

It is estimated that 500–700 LNs are present in the human body, and in small animals such as mice, there are approximately three dozen (Kawashima et al., 1964; Van Den Broeck et al., 2006; Pabst, 2007). LNs are equipped with a tissue architecture suitable for two interdependent functions, i.e., filtrating lymph fluid and inducing immune responses. LN tissue is roughly divided into the cortex and medulla. The cortex is characterized by a high-density lymphocyte accumulation with lymphoid follicles, while the medulla shows a relatively low cell density and intricate network of blood vessels and lymphatic sinuses (Gretz et al., 1997; Ohtani and Ohtani, 2008; Sainte-Marie, 2010). In general, the cortex is thought to be the place where adaptive immune responses are initiated, while the medulla, in which macrophages and other innate immune cells are abundant, is assumed to be the place of innate responses to capture and process lymph-borne substances (Kastenmuller et al., 2012; Qi et al., 2014). Although the LN medulla was known as a typical reticuloendothelial tissue (Hoefsmit et al., 1980; Sakuma et al., 1981; Gordon et al., 2014; Zheng et al., 2016), its physiological function has long been unclear owing to its highly complicated histological organization.

In mice, LNs are much smaller than those of humans and exhibit a relatively simple and regular structure, especially under specific pathogen-free (SPF) conditions. Although the basic features are similar to those of humans, mouse LNs have a relatively large cortex but a narrow medulla, each biasing one side of the organ (Kowala and Schoefl, 1986; Sainte-Marie, 2010). Therefore, the whole tissue structure shows bipolar, with the cortex located on the afferent lymphatic side and the medulla on the efferent side; in this sense, LN medulla in mice is not the literally of “medulla”. Since the structure and function of the medulla in LNs are quite variable depending on the genetic background and immunological burden, its quantitative evaluation is particularly difficult in humans. In contrast, mouse LNs with a relatively simple tissue arrangement are beneficial for investigating their basic principles and physiological roles in the lymphatic system.

The lymph filtering function of LNs is likely to depend on the architecture of lymphatic sinuses, which are the luminal spaces constructed by lymphatic endothelial cells (LECs) (Lammermann and Sixt, 2008; Ohtani and Ohtani, 2008; Gordon et al., 2014). The lymphatic sinuses in LNs are divided into the subcapsular sinus (SS) and medullary sinus (MS), in which different types of macrophages with distinct properties localize, respectively (Gray and Cyster, 2012; Gordon et al., 2014). The afferent lymphatic vessels connected to the LN open into a narrow SS space that expands laterally just below the capsule. A fraction of foreign substances in the lymph fluid is captured by SS macrophages (SSMs) residing at the floor LEC layer of the SS (Phan et al., 2009; Cyster, 2010; Gordon et al., 2014). These SSMs show relatively weak phagocytic activity and transport particulate lymph components to the follicular parenchyma without degradation, thereby playing an important role in B cell responses for antibody production. In contrast, MS macrophages (MSMs) accumulate in the MS, where they express a variety of pattern recognition receptors to capture a wide variety of substances and exhibit strong phagocytic/digestive abilities (Martens et al., 2006; Phan et al., 2009). The medullary cords (MCs), a sheath-like parenchymal structure surrounding the blood vessels, harbors MC/parenchymal macrophages (MC/PMs). The nature of these medullary macrophages is less clear than that of SSMs. Moreover, the extent that the contribution of these sinus structures and macrophage subsets in the LN filtering function is not fully understood.

Multiple LNs are intermittently arranged in key positions in the lymphatic basin drained from a single body site (Kawashima et al., 1964; Harrell et al., 2008; Forster et al., 2012). These draining LNs serve as a multi-step barrier: the “LN chain” system, to remove foreign substances and prevent them from entering the bloodstream. In humans, the flow pathway of lymphatic fluid and the number of LNs vary among individuals, whereas laboratory mice have a relatively simple anatomical pathway consisting of a few LNs. For example, lymph fluid originating in the hind footpad of mice passes through at least three LNs, the popliteal, iliac, and renal nodes, and finally returns to the blood circulation at the subclavian vein (Kawashima et al., 1964; Harrell et al., 2008; Zhang et al., 2013). However, such a stepwise filtration of LNs to prevent foreign substances from entering the blood has not been quantitatively evaluated. In addition, few analyses on the filtering capacity of macrophage subsets in individual LNs have been conducted.

In this study, we examined the lymphatic transport and filtering of subcutaneously injected fluorescent tracers in mice to investigate the histo-anatomical basis and functional significance of LNs as lymph fluid filters. We found a unique organized structure of the lymphatic sinus with a reticular endothelium in a limited area of the LN medulla, in which a specialized macrophage subset is localized as the key effector. We also demonstrated stepwise filtration of the LN chain in the lymphatic basin, which prevents lymph-borne substances from leaking into the blood. These findings indicate that the infrastructure of individual LNs and their systematic orchestration in the LN chain construct a functional gatekeeper system for lymphatic recirculation.

## Materials and Methods

### Mice

C57BL/6JJcl mice were purchased from CLEA Japan. B cell-deficient μMT mice (B6.129S2-*Ighm* ^*tm1Cgn*^/J) have been described previously (Kitamura et al., 1991). Mice lacking peripheral LNs (LN-less) were produced by the intravenous injection of 100 μg LTβR-Fc chimeric protein into pregnant mice on day 12–14 of gestation (Rennert et al., 1996; Katakai et al., 2008). Chimeric protein was purified from culture supernatants or ascites fluid of X63.653 myeloma cells stably transfected with the LTβR-Fc expression vector using a protein A Sepharose column (GE Healthcare). Mice were maintained and crossed under SPF conditions at the animal facility of Niigata University. Mice aged 8–12 weeks were used in the experiments. All animal procedures were approved by the Committee on Animal Research at the Niigata University.

### Subcutaneous Injection of Fluorescent Tracers

Fluorescein sodium salt was purchased from Sigma-Aldrich. Dextran (Dex)-FITC (70 kDa), LPS-AF488, fluorescent latex beads (FluoSphares, 0.1 µm, yellow-green), and *S. aureus*-AF594 were purchased from Thermo Fisher Scientific. OVA (chicken, Sigma) and zymosan A (Wako) were labeled with FITC (Pierce, 46,110). Briefly, OVA (10 mg/ml) or zymosan (20 mg/ml) was dissolved in 0.5 mg/ml FITC, 10 mM Na_2_CO_3_, 100 mM NaCl (pH8.0) and incubated at 37°C or room temperature for 1 h. The FITC-labeled OVA was dialyzed in PBS. FITC-labeled zymosan was washed five times with PBS by centrifugation and stored at −20°C until use.

Fluorescent tracers were subcutaneously injected into the fore footpads, hind footpads, shoulders, chest, flanks, lower abdomens, and tail bases, or alternatively only to the left hind footpad, or left tail base for analyzing individual LNs in a lymphatic basin from single-site drainage. LNs were excised 1 h after injection of fluorescent tracers.

### Whole Lymph Node Microscopy

Excised LNs were soaked in PBS and examined using an inverted microscope CKX41 equipped with a fluorescence observatory unit (Olympus). Digital images were captured using WRAYCAM-NEO1600 or WRAYCAM-NOA2000 (WRAYMER) and processed using Adobe Photoshop CS6 (Adobe Systems) or Affinity Photo (Serif).

### Antibodies

The fluorochrome- or biotin-conjugated, or unconjugated primary antibodies used were as follows: anti-CD3e (145-2C11), anti-B220 (RA3-6B2), anti-CD11c (N418), anti-CD169 (3D6.112), anti-F4/80 (BM8), anti-CD206 (MR6F3), anti-CD45 (30-F11), anti-CD31 (390), and anti-podoplanin (8.1.1) (eBioscience); anti-desmin (rabbit polyclonal) (Abcam); anti-LYVE-1 (BAF2125, goat polyclonal) (R&D Systems); anti-laminin (rabbit polyclonal) (LSL); anti-CD11b (M1/17) (Immunotech); MOMA-2, anti-CD169 (MOMA-1) (Serotec); anti-SIGNR1/CD209b (ER-TR9), anti-F4/80 (BM8), anti-MARCO (ED31) (BMA); anti-FcγRII/III (2.4G2, hybridoma culture supernatant). For secondary reagents, PE-, APC-, Alexa Fluor 488-, AF546-, AF555-, AF594-, or AF633-conjugated streptavidin, anti-rabbit IgG, anti-rat IgG, and anti-goat IgG were purchased from Molecular Probes.

### Fluorescence Immunohistochemistry

Isolated LNs were fixed with 0.05% phosphate buffer containing 0.075 M L-lysine (pH 7.4), 0.01 M NaIO_4_, and 1% paraformaldehyde (PLP fixative) at 4°C for 16–24 h. After fixation, LNs were equilibrated gradually with 10%, 20%, and 30% sucrose in PBS at 4°C, embedded in OCT compound (Sakura Finetech), and frozen at −80°C. Frozen sections (10 µm) were prepared using a cryostat (Leica Biosystems) and post-fixed with cold acetone for 3 min. Sections were stained with antibodies and mounted with Permafluor mountant (Thermo Fisher Scientific). The specimens were examined using an LSM710-NLO confocal microscope (Carl Zeiss), FV1200 confocal microscope (Olympus), or BZ-X800 fluorescence microscope equipped with structured illumination microscopy (SIM) module (Keyence). Digital images were prepared using ZEN (Carl Zeiss), FV10-ASW (Olympus), Adobe Photoshop CS6 (Adobe Systems), and Affinity Photo (Serif).

The longitudinal fluorescent intensity profile and mean fluorescent intensity of the region of interest (ROI) were measured using ImagePro Plus (Media Cybernetics). The fluorescent density was determined by dividing the mean fluorescent intensity by the ROI area. Graphs were created using Microsoft Excel and GraphPad Prism 6.

### Whole-Mount Immunohistochemistry

Excised LNs were fixed with 1% paraformaldehyde in PBS overnight at 4°C, and endogenous peroxidase activity was blocked with 0.3% H_2_O_2_ and 40% methanol in PBS overnight at 4°C (Bogdanova et al., 2018). The specimens were permeabilized with 0.3% Triton X-100 in PBS at 4°C for 1.5 h, followed by blocking with 1% BSA in PBST (0.05% Tween-20 in PBS) at 4°C for 2 h. The specimens were then incubated with 2 μg/ml anti-MARCO antibody (ED31) in 1% BSA in PBST overnight at 4°C and detected using the Vectastain ABC kit and DAB substrate kit (Vector Laboratories).

### Cell Isolation and Flow Cytometry

Single-cell suspensions were prepared from excised LNs, cut into small fragments, and digested with 1 mg/ml collagenase D and 0.1 mg/ml DNase I (Roche Diagnostics) as described (Takeuchi et al., 2018), followed by counting the total cell numbers using a hemocytometer. Isolated cells were stained with fluorescently labeled antibodies. Data were acquired using a FACSCalibur flow cytometer (BD Biosciences) and analyzed using CellQuest (BD Biosciences) and FlowJo. Dead cells were excluded by forward scatter and propidium iodide staining.

The total uptake of Dex-FITC in a given cell population or each LN was calculated by multiplying the total cell number by the mean fluorescence intensity. The total Dex-FITC uptake in the lymphatic basin is the sum of the total uptake in each LN.

### Macrophage Depletion

Macrophages in the LNs were depleted by the subcutaneous injection of clodronate liposomes (CLL) (Hygieia Bioscience) into the footpads, shoulders, chest, flanks, and backs, or alternatively, only to the left hind footpad (100 µg/site), 48 h before Dex-FITC injection. For the restricted depletion of macrophages in the popliteal LN, 0.3 µg CLL was injected into the left hind footpad.

### Lymphadenopathy Induction

Lymphadenopathy in the popliteal LN was induced by the injection of 1 mg/ml OVA in a 1:1 emulsion of PBS and complete Freund's adjuvant (CFA) (Wako) into the instep of the left hind foot, and 7 days later Dex-FITC was injected into the footpad.

### ELISA

Dex-FITC in the sera was measured using a custom-built ELISA. Ninety-six well plates were coated with anti-FITC monoclonal antibody (1F8-1E4, Invitrogen) at 4°C overnight and blocked with 1% bovine serum albumin (BSA), 0.05% Tween 20, PBS. Mouse sera were 1/10 diluted with PBS and added to the plate and incubated at room temperature for 2 h. After washing with PBST, the plate was incubated with a biotin-conjugated anti-FITC monoclonal antibody (NAWESLEE, eBioscience) for 1 h, followed by streptavidin-horseradish peroxidase (R&D systems) for 20 min. TMB substrate reagent (R&D systems) was then added to each well and the plate was incubated for a further 30 min; 2N H_2_SO_4_ solution was added to stop the reaction. The absorbance at 450 nm was measured using a Multiskan FC multiplate photometer (Thermo Fisher Scientific), and the basal absorbance at 570 nm was subtracted from the values.

### Statistical Analysis

Microsoft Excel and GraphPad Prism 6 were used for statistical analyses. The means of the two groups were compared using unpaired Student’s *t*-tests. Mann-Whitney *U*-tests were used to compare two nonparametric groups and one-way analyses of variance (ANOVA) with Tukey’s multiple comparisons tests when comparing more than two groups. Significance was set at *p* < 0.05.

## Results

### Lymph-Borne Substances Are Accumulated in a Limited Area of the Lymph Node Medulla

**FIGURE 1:**
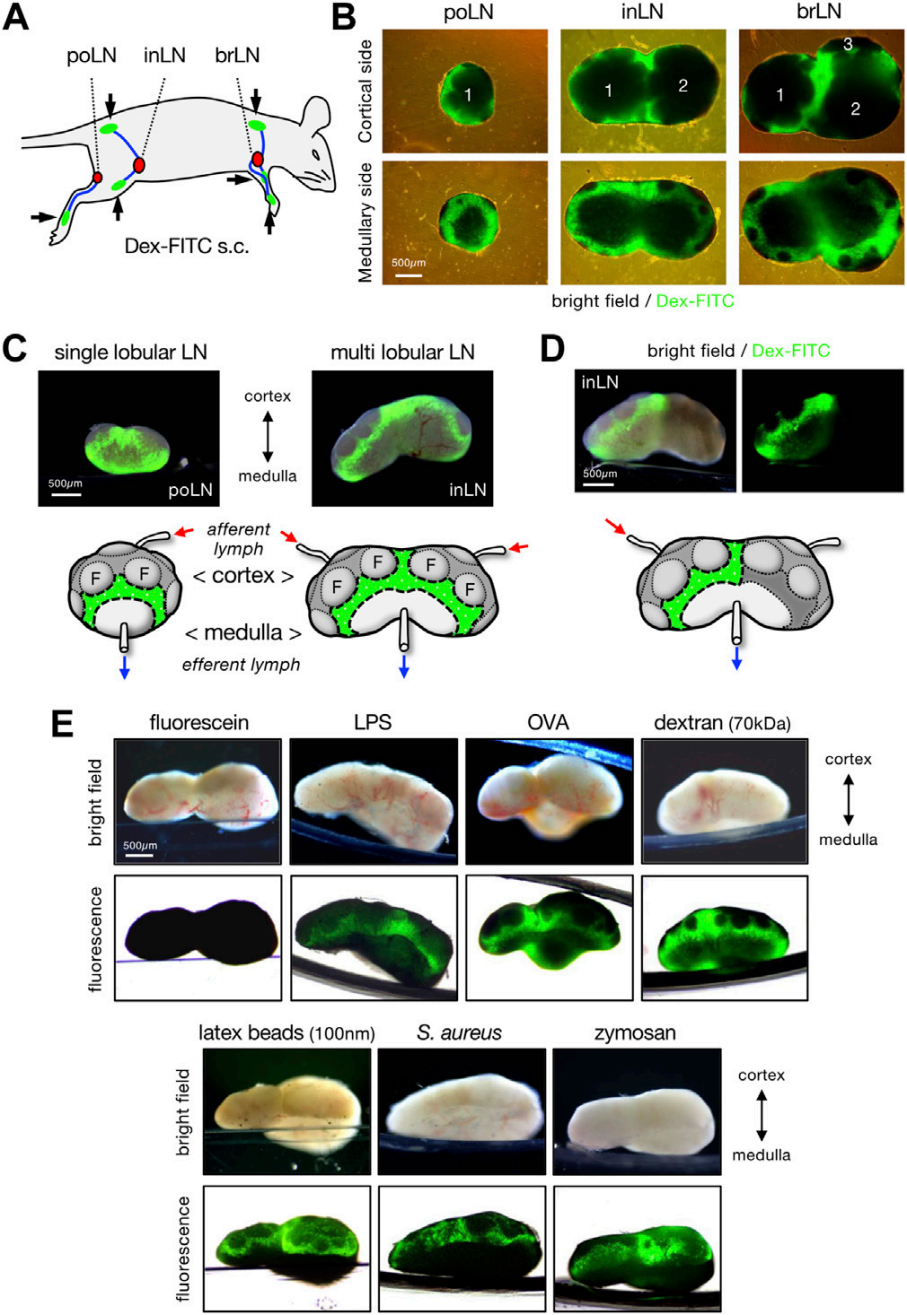
Lymph-borne substances are accumulated in a limited area of LN. **(A)** Schematic representation of the subcutaneous injection sites (arrows and green areas) of fluorescent tracer and lymphatic drainage routes (blue lines) to the regional draining LNs (red nodules). brLN, brachial LN; inLN, inguinal LN; poLN, popliteal LN. **(B)** Whole organ appearance of LNs that accumulate Dex-FITC. LNs were excised 1 h after the subcutaneous Dex-FITC injection. Composite of transmission and fluorescent images from the cortical side (upper) and the medullary side (lower) of LNs are shown. The numbers represent the composition of lobules. **(C)** Lateral views of single lobular (popliteal) and multi lobular (inguinal) LNs. Brightfield/fluorescent composite images (upper) and schematic representations that show the area of Dex-FITC accumulation (lower) are shown. Small arrows indicate the direction of lymphatic flow. F, follicle. **(D)** Lateral view of the inguinal LN that received the lymphatic drainage of single site (tail base) Dex-FITC injection. **(E)** Lateral views of the brachial LNs that received various fluorescent tracers. LNs were excised 1 h after subcutaneous tracer injection. Brightfield (upper) and transmission/fluorescent composite (upper) images are shown.

To evaluate the LN filtering function, mice were subcutaneously injected with 70 kDa Dex-FITC and skin-draining LNs were removed 1 h later for examination of the overall organ appearance by fluorescence microscopy (Figures 1A,B). In single-lobular LNs, such as the popliteal LNs, a belt-like accumulation of Dex-FITC was observed in the lateral side of the organ near the border of the medulla and cortical lymphoid follicles (Figures 1B,C). In multi-lobular inguinal or brachial LNs, similar Dex-FITC accumulation was detected around the cortex–medulla border, especially where the two lobules were adjoined (Figures 1B,C, Supplementary Figure S1). In general, individual lobules in LN receive lymph fluid drained from specific parts of the body. When Dex-FITC was injected to the tail base, the accumulation was detected only on one side of the lobules in the inguinal LN, in which the lymph from the lower abdomen or the tail base drains either of the lobules (Figures 1A,D). This suggests that the lymph filter in each lobule functions independently.

In addition to dextran, relatively small-sized soluble components such as lipopolysaccharide (LPS, >100 kDa) and ovalbumin (OVA, 43 kDa) conjugated with fluorescent dyes or 100 nm latex beads showed similar accumulation, whereas larger particulate substances such as bacteria *S. aureus* (0.5–1 µm) and zymosan (∼3 µm) tended to accumulate more in slightly cortical parts than in the cortex–medulla border (Figure 1E). However, soluble fluorescein (Mw 332) did not show any accumulation, although we observed fluorescence in the urine 1 h after injection, indicating that the injected fluorescein had passed through the LNs. This suggests that the capture of lymph-borne substances in this part of LNs requires molecular signatures or sizes independent of FITC residue. At present, the minimum sized molecule detectable by the accumulation in LNs is soluble OVA protein (∼6 nm).

### Lymph-Borne Substances Accumulate in a Specialized Part of the MS

**FIGURE 2:**
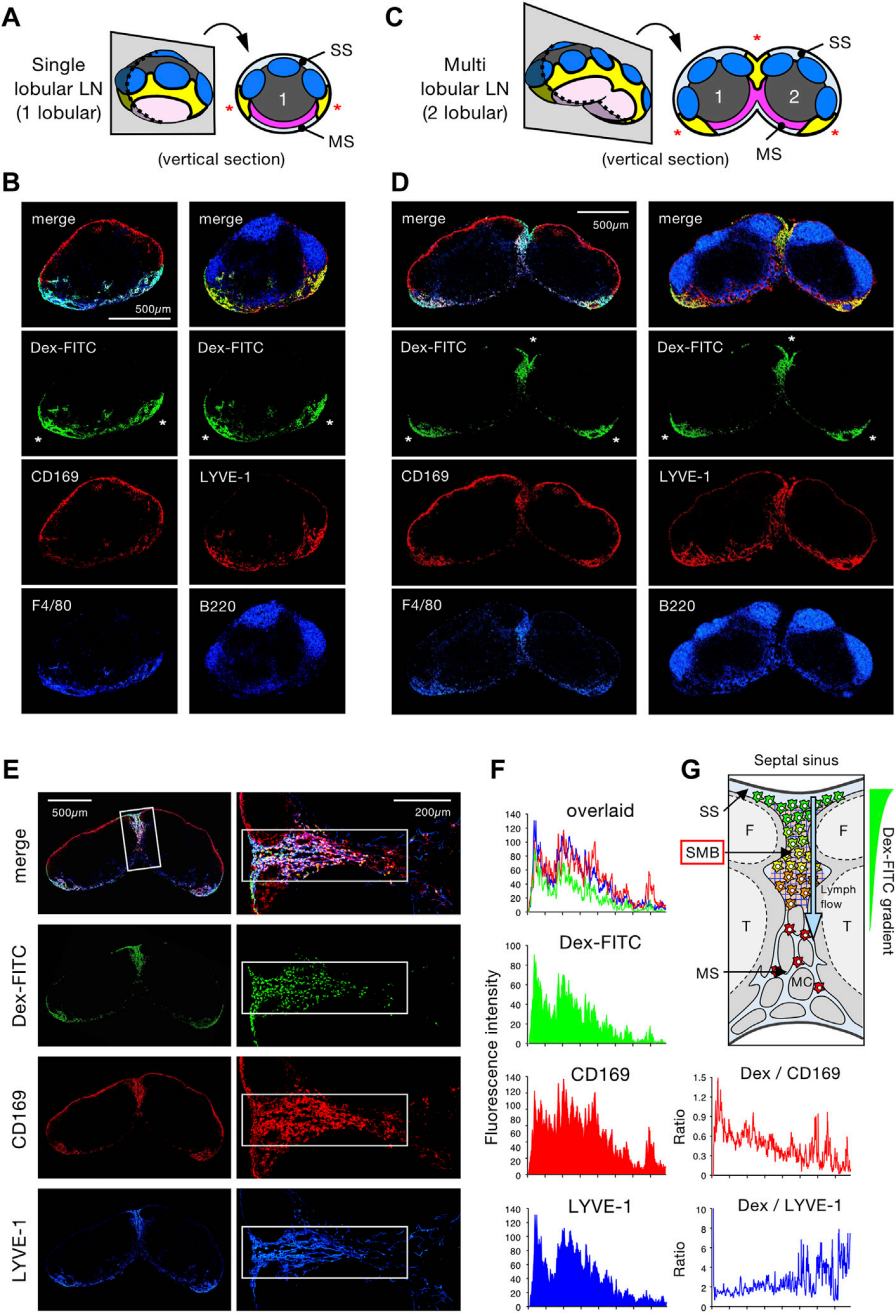
Lymph-borne substances accumulate in the limited areas of the LN medullary sinus. **(A–D)** Confocal immunohistochemical examinations of Dex-FITC–drained LNs. Schematic representations of the tissue sectioning for single lobular (popliteal) LN **(A)** and multi lobular (inguinal) LN **(C)**, in which the numbers indicate lobular composition and asterisks indicate the arias of Dex-FITC accumulation in the tissue sections (yellow parts). Confocal immunohistochemical images of the popliteal **(B)** and inguinal **(D)** LNs. Dex-FITC accumulation (asterisks) with the indicated antibody staining are shown. MS, medullary sinus; SS, subcapsular sinus. **(E)** Confocal immunohistochemical images of the inguinal LN (left) and close-up view of the septal MS between two lobules (right). **(F)** Quantitative image analysis of the septal MS. Average fluorescent intensity profiles along with the cortex-medulla axis of the rectangular boxed region in **(E)**. **(G)** Schematic diagram showing the physical barrier of the subcapsular-medullary sinus border (SMB) region in the septal MS with an emphasis on macrophages in the sinus and the gradient of internalized Dex-FITC. F, follicle; MC, medullary cord; T, T cell area.

To examine the histological details of Dex-FITC accumulation in draining LNs, we performed immunostaining on tissue sections followed by confocal microscopy. The longitudinal section of single-lobular LNs shows that multiple follicles are centered on a paracortical T-cell area in the cortical hemisphere, while the MS and MCs are concentrated in the limited area of the medullary hemisphere **(** Supplementary Figure S1 **)**. Dex-FITC constantly accumulated on the medullary side of the border between the SS and MS, in which CD169^+^F4/80^+^ MSMs were concentrated (Figures 2A,B). This part coincided with a LYVE-1^high^ area, suggesting the presence of intricate lymphatic sinuses. On the other hand, Dex-FITC was barely detected in the conduit because the size of the dextran used (70 kDa) is known to be excluded from this structure (Gretz et al., 2000). In the section of multi-lobular LNs, two adjacent lobules are connected to form a joint MS that created a septum of the lobules (septal MS) **(** Supplementary Figure S1). As in single-lobular LNs, the accumulation of Dex-FITC was found on the medullary side of the border, however, there were three net FITC^+^ areas in the whole-organ sections, where CD169^+^F4/80^+^ MSMs and LYVE-1^+^ LECs were also concentrated (Figures 2C,D). We also examined LNs that received subcutaneously injected *S. aureus*. However, consistent with Figure 1E, relatively large-sized particulate components such as bacteria were inefficient in reaching the LNs, and even if they did enter, they were mainly retained in the SS. (Supplementary Figure S2).

Detailed examinations revealed two subregions in the MS: areas where Dex-FITC accumulates or not (Supplementary Figure S3 **)**. Dex-FITC accumulation was selectively observed in the region closer to the cortex, in which LYVE-1^+^ LECs were concentrated to form an interconnected network. These are sinusoidal reticular cells; however, we refer to them more directly as reticular LECs (rLECs). MSMs were basically colocalized with this rLEC network (Supplementary Figure S3C **)**. In contrast, the sinuses in the deeper medulla showed a typical tubular shape composed of flat LECs with few macrophages in the lumen, in which Dex-FITC was poorly accumulated. The flat LECs constituted the layer surrounding the MCs that harbored numerous MC/PMs with little Dex-FITC uptake. Therefore, the LN filter that captures Dex-FITC is confined in a particular part of the MS, and we propose to call it the subcapsular-medullary sinus border (SMB).

### MSMs Localized in the Subcapsular-Medullary Sinus Border Are Responsible for the Lymph Node Filter Function

The SMB possibly acts as a physical barrier for removing lymph-borne substances. To test this, we examined septal MS in multi-lobular LNs, in which Dex-FITC was accumulated. By quantitatively analyzing the fluorescence intensity, a clear gradient of FITC signal was observed from the top of the septal MS adjacent to the SS to the bottom, while the distribution of the CD169 signal expressed by MSMs was relatively uniform (Figures 2E–G). The ratio of FITC to CD169 signals that represents Dex-FITC uptake per macrophage showed a significant gradient, while no gradient was detected in the ratio of FITC to LYVE-1, indicating that the FITC/CD169 gradient does not depend on sinus width. Therefore, MSMs localized upstream of the SMB captured more Dex-FITC than in the downstream, suggesting that the SMB acts as a physical barrier for lymph filtering.

**FIGURE 3:**
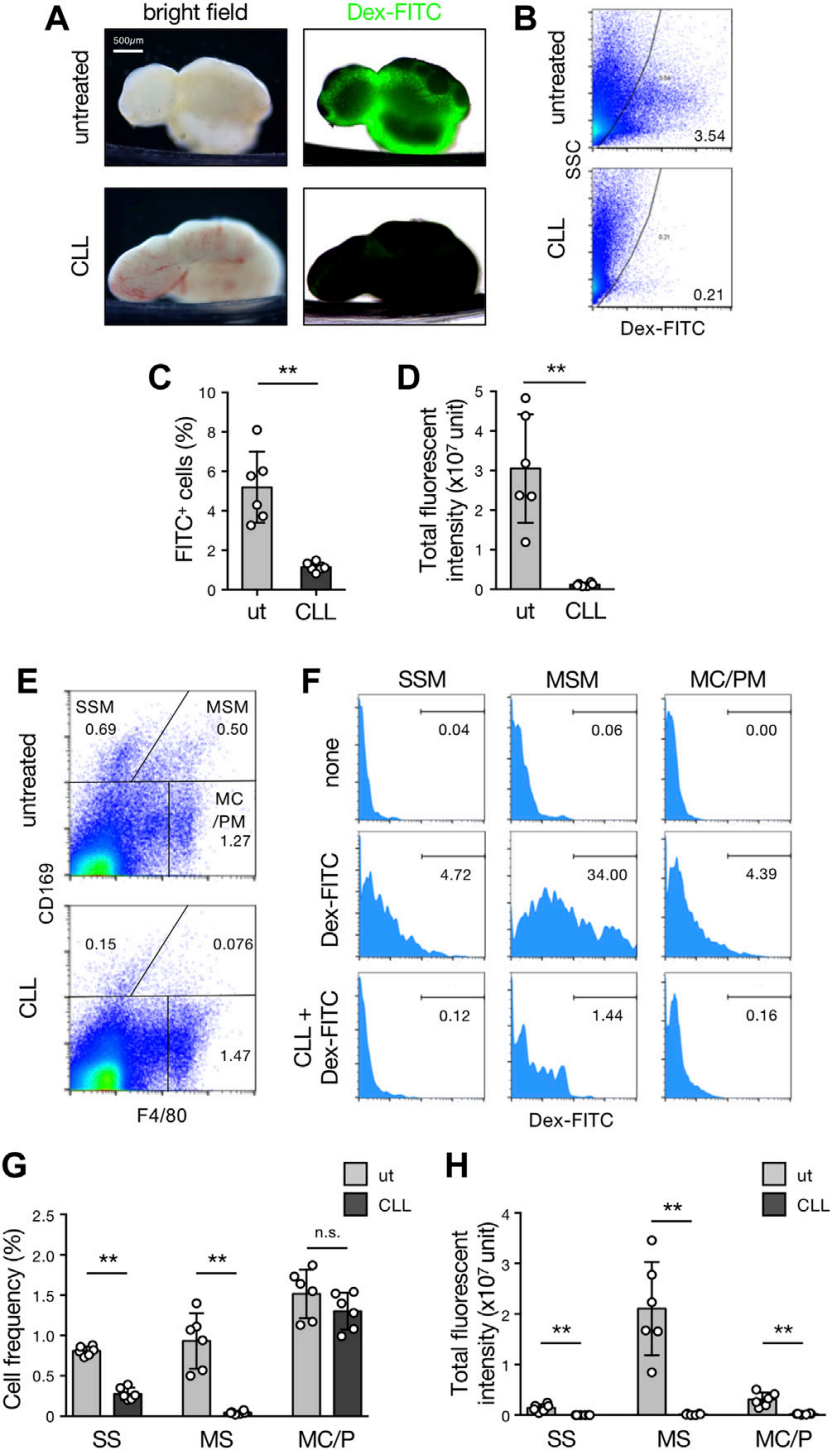
MSMs localized in the SMB are responsible for the LN filter. **(A)** Brightfield (left) and transmission/fluorescent composite (right) images of the brachial LNs with or without subcutaneous CLL pretreatment received the drainage of the Dex-FITC injection. **(B–D)** Flow cytometric analysis for detecting cells that engulfed Dex-FITC in total LN cells. Mice with or without subcutaneous CLL pretreatment were injected with Dex-FITC (20 µg/site) and brachial, inguinal, and popliteal LNs were excised 1 h later to isolate cells by enzymatic digestion. **(B)** Representative dot plots of flow cytometry. Cells in the right side of the curved line are regarded as Dex-FITC^+^ and the number indicates the percentage in total live cells. **(C)** The percentages of Dex-FITC^+^ cells in total LN cells. Each open circle indicates the result of an individual mouse. Mean ± SD. **p < 0.005. ut, untreated. **(D)** Total fluorescent intensities calculated from the mean fluorescent intensity (MFI) and total cell number. Mean ± SD. **p < 0.005. **(E,F)** Identification of macrophage subset that engulf Dex-FITC in LNs. **(E)** Flow cytometric fractionation of macrophage subsets in LNs by the expression of CD169 and F4/80. Representative dot plots are shown. SSM, subcapsular sinus macrophage; MSM, medullary sinus macrophage; MC/PM, medullary cord/parenchymal macrophage. **(F)** Histograms for Dex-FITC in each macrophage subset gated in **(E)**. The number indicates the percentage of Dex-FITC fraction. **(G)** The percentages of macrophage subsets in LNs. Mean ± SD. **p < 0.005. n. s., not significant. **(H)** Total fluorescent intensities in macrophage subsets. Mean ± SD. **p < 0.005.

We next tested the Dex-FITC accumulation after the depletion of phagocytes by subcutaneous injection of CLL. In the LNs of untreated mice, Dex-FITC accumulation was prominent in the SMB and many FITC^+^ cells were detected in isolated LN cells by flow cytometry (Figures 3A–D). In contrast, the FITC signal in the whole organ was nearly eliminated by macrophage depletion, and FITC^+^ cells were dramatically decreased in flow cytometry. LN macrophages can be fractionated into three populations by the expression of CD169 and F4/80: SSM (CD169^+^F4/80^−^), MSM (CD169^+^F4/80^+^), and MC/PM (CD169^–^F4/80^+^). Among them, Dex-FITC uptake was remarkably concentrated in the MSM fraction (total fluorescent intensity of 82.3% ± 1.4% in macrophages and 69.6% ± 2.8% in LN cells) (Figures 3E–G). Subcutaneous CLL injection significantly reduced the number of SSMs and MSMs but had little effect on MC/PMs (Figure 3G, Supplementary Figure S4A), indicating that macrophages in the sinus lumen were selectively removed. CLL eliminated FITC^+^ fractions in all subsets, especially in MSMs, thereby dramatically reducing the Dex-FITC uptake in LNs (96.2% reduction) (Figure 3H).

B cell-deficient µMT mice lack SSMs but not MSMs (Moseman et al., 2012). However, lymph-borne Dex-FITC was clearly accumulated in the lateral part of the organ (Supplementary Figure S5A). Flow cytometric analysis showed that the numbers of MSMs and MC/PMs, except SSMs, and total Dex-FITC uptake in µMT LNs were comparable to that in wild-type mice (Supplementary Figures S5B–G). This suggests that the contribution of SSMs to the LN filter is negligible. Collectively, MSMs localized in the SMB are responsible for the main body of lymph filtering function in LNs.

### MARCO^+^ MSMs and Reticular LECs Construct the Subcapsular-Medullary Sinus Border

**FIGURE 4:**
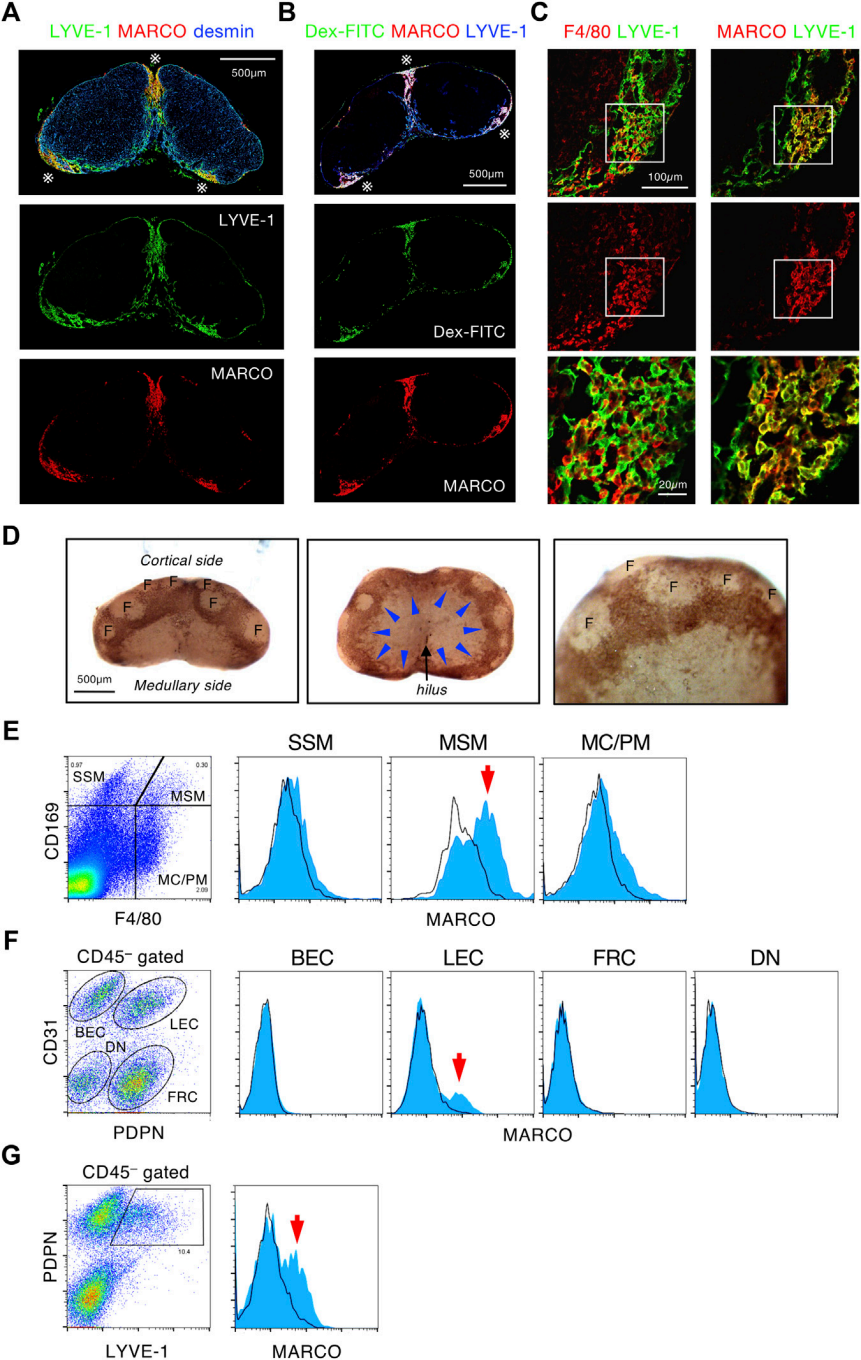
MARCO^+^ MSMs and reticular LECs construct the SMB. **(A,B)** MARCO is a highly specific marker of the SMB region. **(A)** Confocal immunohistochemical images of the inguinal LN stained with MARCO, LYVE-1, and desmin. Asterisks indicate the SMB. **(B)** The inguinal LN from Dex-FITC injected mouse was stained with MARCO and LYVE-1. **(C)** Both macrophages and LECs express MARCO in the SMB. Close up view of the SMB with LYVE-1^+^ LECs shows reticular structure. The boxed region in the upper panels is shown in the bottom panels as higher magnification views. **(D)** Whole mount immunohistochemical staining of MARCO in the inguinal LN. Lateral (left) and medullary (middle) views of the whole LN and higher magnification view (right). Arrowheads indicate band-like MARCO^+^ area (brown) corresponding to the SMB. F, follicle. **(E)** Flow cytometric analysis of MARCO expression in macrophage subsets. Cells from enzymatically digested LNs are fractionated by CD169 and F4/80 expression, and MARCO expression (blue) in each macrophage subsets is shown in the right histograms. Black line shows isotype control. Arrow indicates MARCO^+^ cells detected in MSM fraction. **(F,G)** Flow cytometric analysis of MARCO expression in stromal cell subsets. CD45^–^-gated cells isolated from enzymatically digested LNs are fractionated by the expression of CD31/podoplanin (PDPN) **(F)** or PDPN/LYVE-1 **(G)**. MARCO expression in each stromal cell subsets is shown in the right histograms. BEC, blood endothelial cell; LEC, lymphatic endothelial cell; FRC, fibroblastic reticular cell; DN, double negative. Arrows indicate MARCO^+^ cells detected in LEC fractions.

To further explore the characteristics of the SMB, we examined the various markers of macrophages and LECs in tissue sections. This screening revealed that the scavenger receptor MARCO was strongly and selectively expressed in the SMB (Supplementary Figure S6A, Figures 4A–C). In addition, the whole-mount immunostaining demonstrated that MARCO staining clearly highlighted the band-shaped SMB (Figure 4D). Flow cytometric analysis confirmed that a fraction of MSMs expressed MARCO among macrophage subsets (Figure 4E). However, LYVE-1^+^ LECs in the SMB also appeared to be stained with MARCO (Figure 4C). To confirm this, we fractionated LN stromal cells by flow cytometry and found significant MARCO expression in a fraction of CD45^−^CD31^+^Pdpn^+^ LECs (Figure 4F). In LYVE-1^+^ LEC fraction, we detected an even higher MARCO^+^ population (Figure 4G). CLL treatment depleted MARCO^+^ MSMs but not MARCO^+^ rLECs in the SMB (Supplementary Figure S4B), suggesting that macrophages are responsible for lymph filtering function, although the structural alteration of SMB by macrophage depletion may have some influence on this. Consistent with these findings, recent single-cell RNAseq analysis of the LEC fraction in LNs has shown the presence of a MARCO^+^ subset in the region corresponding to the SMB (Xiang et al., 2020). Therefore, SMB is a unique area characterized by the expression of MARCO in both MSMs and rLECs.

### The Lymph Node Chain Acts as a Filtering System to Remove Lymph-Borne Substances

**FIGURE 5:**
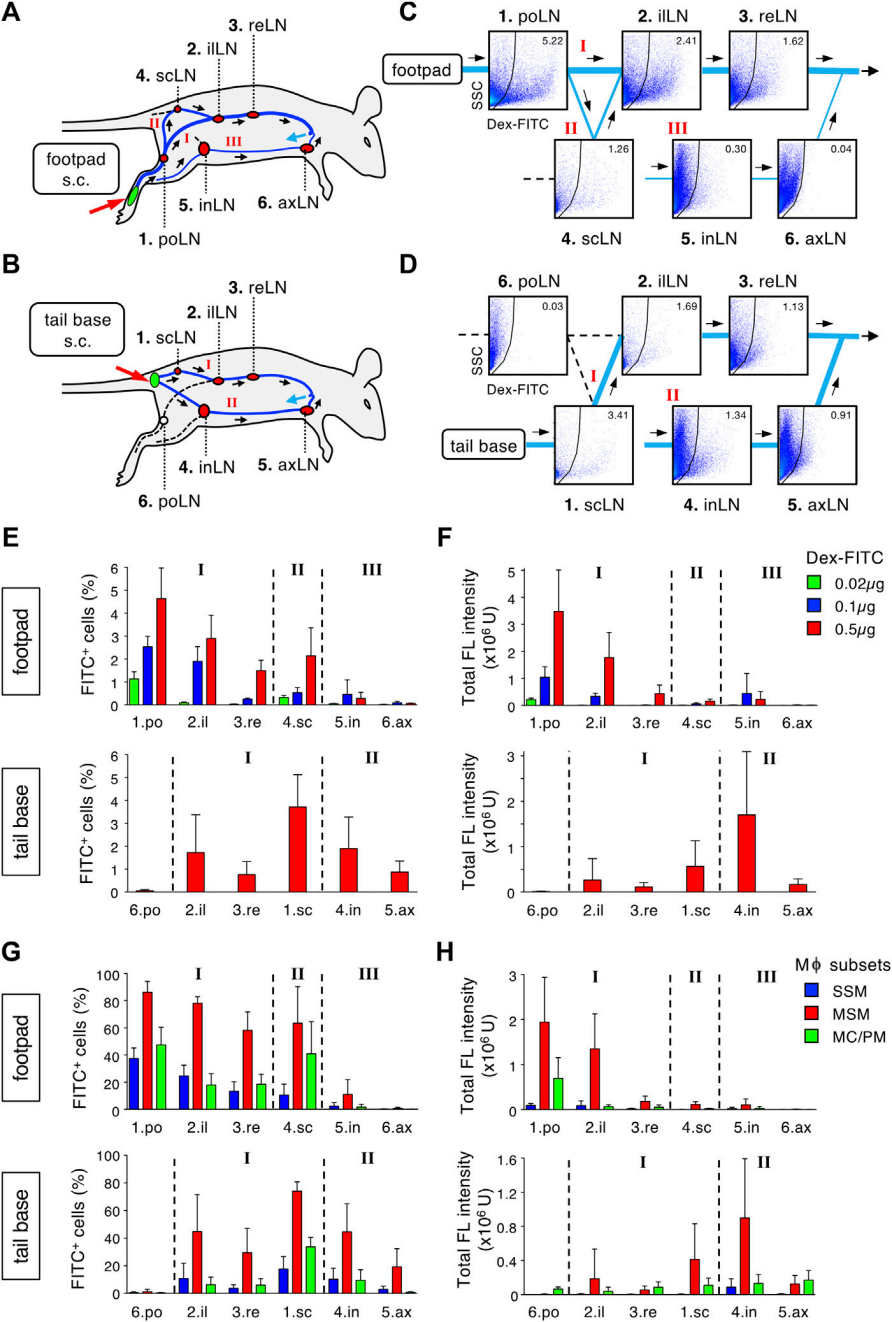
The LN chain in the lymphatic basin acts as a stepwise filter to remove lymph-borne substances. **(A,B)** Schematic representation of the lymphatic route drained from a single site of subcutaneous tracer injection and intervening LNs. Dex-FITC that is injected into the hind footpad **(A)** or tail base **(B)** drains *via* lymphatic vessels and passed through several LNs in a route dependent manner. There are some main, roundabout, and alternative routes indicated by roman numerals. po, popliteal; il, iliac; re, renal; sc, sciatic; in, inguinal; ax, axillary. **(C,D)** Systematic flow cytometric analysis for detecting Dex-FITC^+^ cells in individual LN in the lymphatic basin. Mice were subcutaneously injected with 0.5 µg Dex-FITC into the hind footpad **(C)** or tail base **(D)**. Representative set of dot plots are placed at appropriate locations on the flow chart of the lymphatic drainage route corresponding to **(A)** and **(B)**, respectively. **(E,F)** The percentages of Dex-FITC^+^ cells **(E)** and total fluorescent intensity **(F)** of individual LN in the injection of the hind footpad (upper) or tail base (lower). In footpad injection, three different doses of Dex-FITC (0.02, 0.1, and 0.5 µg) were examined. Mean ± SD. footpad: 0.02 µg, *n* = 3; 0.1 µg, *n* = 3; 0.5 µg, *n* > 5. tail base: 0.5 µg, *n* = 4. **(G,F)** The percentages of Dex-FITC^+^ cells **(G)** and total fluorescent intensity **(H)** of macrophage subsets in individual LN in hind footpad (upper) or tail base (lower) injections. footpad: *n* > 5. tail base: *n* = 4.

Lymph fluid from a certain body part is gathered in lymphatic vessels and finally returns to the bloodstream. During this process, a sequence of LNs is expected to filter foreign substances. To quantitatively evaluate the filtration capacity of the LN chain, we conducted a systematic analysis by which Dex-FITC was subcutaneously injected into one site and individual LN in the lymphatic basin was examined by flow cytometry (Figures 5A,B, Supplementary Figures S7, S8). Starting from the hind footpad, the main route passes through at least three LNs: the first draining popliteal LN, followed by the iliac and renal LNs, after which it joins the subclavian vein through the thoracic duct (Figure 5A, Route I) (Kawashima et al., 1964; Harrell et al., 2008). In addition, a bypass route branches off downstream of the popliteal LN, which passes through the sciatic LN and then rejoins the iliac LN (Figure 5A, Route II**)**. There is another lateral route from the hindlimb to the inguinal region, which drains in the inguinal LN and connects to the axillary LN (Figure 5A, Route III**)**. If Dex-FITC is injected into the tail base, the first draining site is the sciatic LN, followed by the iliac and renal LNs (Figure 5B, Route I). In this case, lateral drainage to the inguinal LN also plays a major role (Figure 5B, Route II**)**, while the popliteal LN does not contribute to this pathway.

Reflecting the lymphatic anatomy, the proportion of FITC^+^ cells in total LN cells was the highest in the first draining LNs, depending on the injection sites, and tended to decrease in LNs of downstream, bypass, and alternative routes (Figures 5C–F, Supplementary Figures S7, S8). When the Dex-FITC dose was reduced, the range of reach in the LN chains was shortened and the total uptake decreased (Figures 5E,F). We determined that the injection dose 0.02 µg/site would detect FITC signal almost exclusively in the popliteal LN. When we examined macrophage subsets in each LN, MSMs showed the highest Dex-FITC uptake (total fluorescent intensity of 79.5% ± 7.7% in macrophages and 62.2% ± 8.2% in LN cells), and the percentage of FITC^+^ cells as well as the total uptake tended to decrease as downstream LNs (Figures 5G,H). Therefore, lymph-borne substances drained from a single body part are filtered by the LN chain in which MSMs play a crucial role.

### Stepwise Filtering by the Lymph Node Chain Prevents Lymph-Borne Substances From Entering the Blood

**FIGURE 6:**
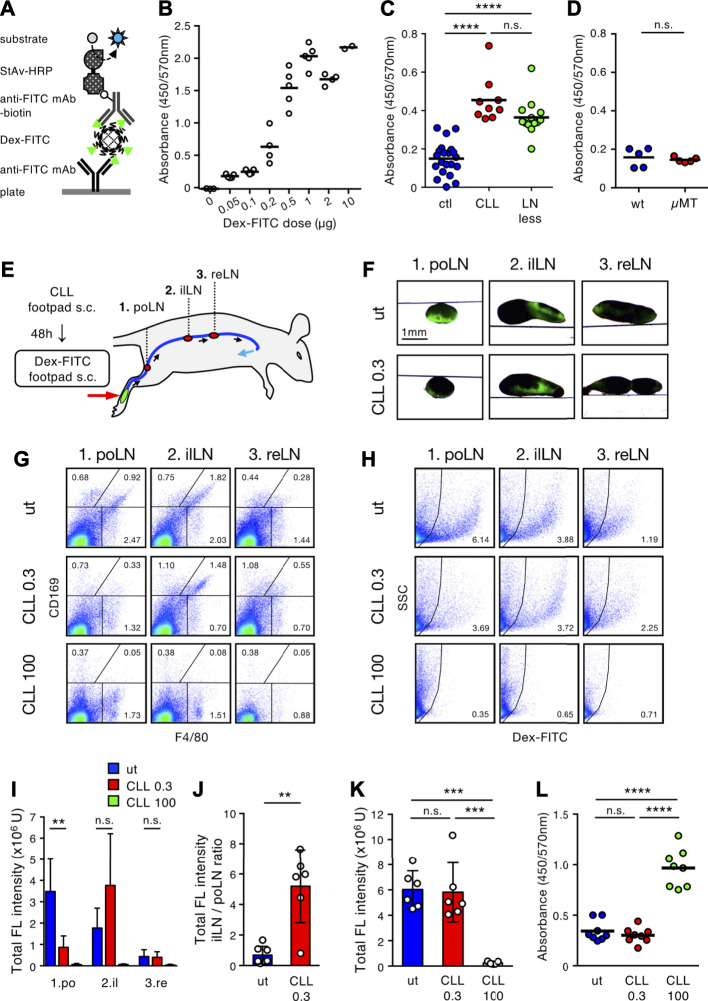
Stepwise filtering of the LN chain prevents lymph-borne substances from entering the bloodstream. **(A)** Schematic diagram of ELISA system for detecting serum Dex-FITC. HRP, horse radish peroxidase; mAb, monoclonal antibody; StAv, streptavidin. **(B)** Injection dose-dependent increase of serum Dex-FITC. Various doses of Dex-FITC were injected into the hind footpad and sera were harvested 1 h later for ELISA detection. Each open circle indicates the result of an individual mouse and bars indicate the mean of the group. **(C)** Serum Dex-FITC detection by the hind footpad injection (0.1 µg) in the control (ctl), subcutaneous CLL pretreatment, and LN-less mice. ****p < 0.0001; n. s., not significant (one-way ANOVA). **(D)** Serum Dex-FITC detection by hind footpad injection (0.1 µg) in wt and µMT mice. n. s., not significant. **(E)** Schematic representation of the experimental setting and the main lymphatic route/intervening LNs drained from the hind footpad. **(F)** Transmission/fluorescent composite images of the popliteal (po), iliac (il), and renal (re) LNs, 1 h after the hind footpad Dex-FITC injection in untreated (ut) or 0.3 µg CLL pretreated mice. **(G,H)** Flow cytometric analysis of macrophage subsets **(G)** and Dex-FITC^+^ cells **(H)** in individual LN with the different doses (0.3 and 100 µg) of CLL pretreatment. **(I,J)** Total fluorescent intensity in individual LN **(I)** and the ratio of total fluorescent intensity of popliteal to iliac LNs **(J)**. ut, *n* = 7; CLL 0.3, *n* = 6; CLL 100, *n* = 5. Mean ± SD. **p < 0.01. n. s., not significant **(K)** Total fluorescent intensity of all LNs in the lymphatic basin. Mean ± SD. ***p < 0.0005; n. s., not significant (one-way ANOVA). **(L)** Serum Dex-FITC detection in the different doses of CLL pretreatment (0.3 and 100 µg). Dex-FITC (0.1 µg) was injected into hind footpad and sera were harvested 1 h later for ELISA. Mean ± SD. ****p < 0.0001; n. s., not significant (one-way ANOVA).

We next attempted to establish a system by which to analyze whether subcutaneously injected Dex-FITC leaks into the blood. One hour after the single-site Dex-FITC injection, serum was collected to measure the content of FITC by ELISA (Figure 6A). Because the increase in serum FITC was clearly detected in a manner dependent on Dex-FITC dose (<1 µg), it is assumed that the leakage of Dex-FITC into the blood could be detected quantitatively within some range (Figure 6B). This also indicates that excessive Dex-FITC in the peripheral lymph above the limit of the capacity of the LN chain allowed it to leak into the blood. Next, mice with CLL pretreatment or lacking most LNs owing to *in utero* LTβR-Fc treatment (LN-less) were injected with 0.1 µg Dex-FITC, an amount with low blood leakage in the control. We detected a significant increase of FITC in the sera from mice treated with CLL and those in the LN-less condition (Figure 6C). In contrast, µMT mice showed no significant difference in Dex-FITC leakage compared with wild-type mice (Figure 6D), suggesting that SSMs are dispensable. Therefore, the sequential filters of the LN chain mediated by MSMs are required for preventing lymph-borne substances from entering the blood.

If the sequence of LNs cooperatively filters lymph fluid in a stepwise manner, functional failure in an upstream LN may lead the downstream LN to compensate for it. To test this, we sought to determine the dose of CLL treatment that depletes MSMs only in the first draining LN, with little or no influence on the downstream LN. Consequently, we found that a dose of 0.3 µg CLL could remove MSMs only in the popliteal LNs **(** Figures 6E–H). In this setting, Dex-FITC uptake in the popliteal LN markedly decreased, while it clearly increased in the iliac LN (7.65-fold increase in iLN/poLN ratio) (Figures 6H–J), indicating that lymph-borne substances that could not be filtered out by the upstream LNs were captured in the downstream LN. However, the overall filtering efficiency of the LN chain was preserved and Dex-FITC leakage into the blood was kept as low as that of the untreated controls (Figures 6K,L). Therefore, stepwise filtering by the LN chain system prevents lymph-borne substances from entering the blood.

### Inflammatory Response Remodels the Lymph Node Chain System and Enhances the Filter Function

**FIGURE 7:**
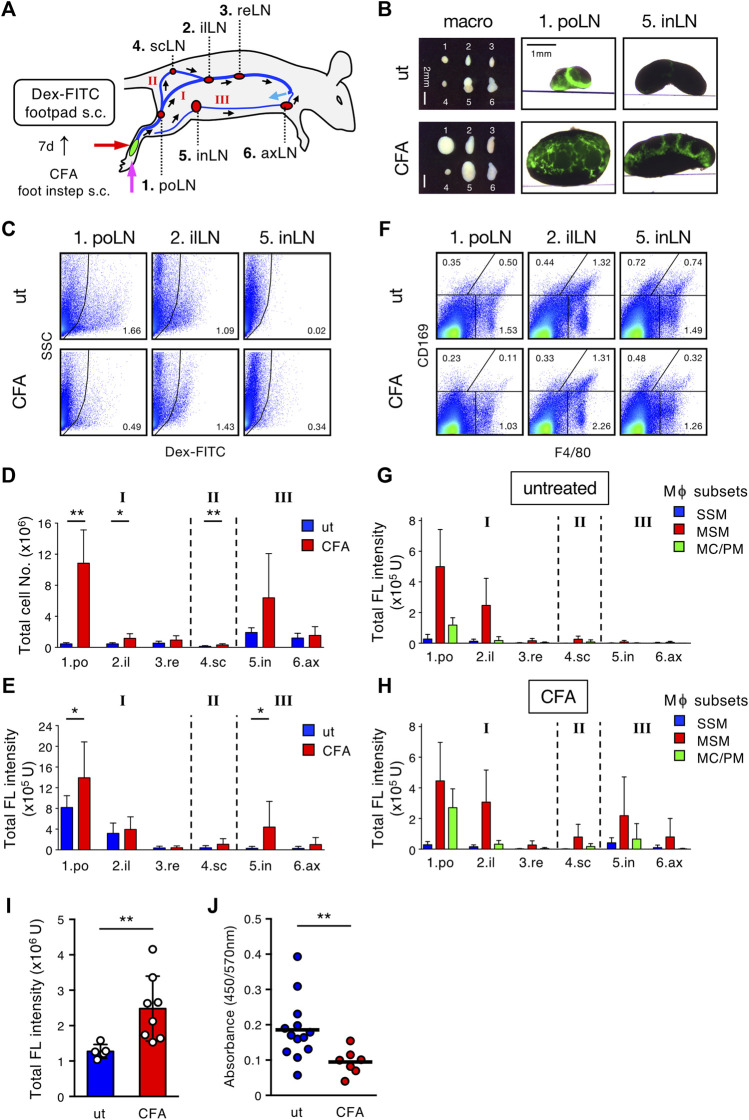
Inflammatory response remodels the LN chain system and enhances the filtering efficiency. **(A)** Schematic representation of the experimental setting and the lymphatic routes/intervening LNs drained from the hind footpad. **(B)** Transmission/fluorescent composite images of the popliteal (po) and inguinal (in) LNs, 1 h after the hind footpad Dex-FITC injection (0.5 µg) in untreated (ut) or CFA (OVA/CFA) pretreated mice. **(C)** Flow cytometric analysis of Dex-FITC^+^ cells in individual LN with or without OVA/CFA pretreatment and Dex-FITC injection (0.1 µg). **(D,E)** Total cell number **(D)** and total fluorescent intensity **(E)** in individual LN. ut, *n* = 5; CFA, *n* = 8. Mean ± SD. *p < 0.05, **p < 0.005. n. s. not significant. **(F)** Flow cytometric analysis of macrophage subsets in individual LN with or without OVA/CFA pretreatment. **(G,H)** Total fluorescent intensity of macrophage subsets in individual LN from untreated **(G)** or OVA/CFA pretreated **(H)** mice. ut, *n* = 5; CFA, *n* = 8. Mean ± SD. **(I)** Total fluorescent intensity of all LNs in the lymphatic basin. Mean ± SD. **p < 0.005. **(J)** Serum Dex-FITC detection in untreated or OVA/CFA pretreatment. Mean ± SD. **p < 0.005.

Finally, we sought to determine whether the lymph filtering of LNs is altered by inflammatory tissue remodeling. CFA containing OVA was administered to the hindfoot instep and 7 days later, Dex-FITC was injected into the footpad (Figure 7A). Compared with controls, mice injected with CFA showed dramatically enlarged popliteal LNs and significant expansion of the downstream LNs (Figure 7B). In hypertrophic popliteal LNs, Dex-FITC did not show clear band-shape accumulation; rather it showed a branched pattern with relatively weak intensity, and filamentous MARCO^+^ MS had penetrated deep into the tissue (Figure 7B, Supplementary Figure S6B). However, Dex-FITC uptake in the popliteal LNs significantly increased (1.49-fold) in CFA treatment, although the percentage of FITC^+^ cells decreased owing to the dramatic increase in total lymphocytes (Figures 7C–E). Interestingly, the inguinal LNs, which showed little uptake in the steady state, exhibited a marked increase in Dex-FITC uptake (Figure 7B, Supplementary Figure S6B), suggesting that inflammation may remodel the lymphatic flow pathway. FITC^+^ macrophages also increased in the inguinal and axillary LNs (Figures 7F–H). Thus, the complex alterations significantly augmented the total uptake of Dex-FITC in the lymphatic basin (1.94-fold), while leakage into the blood clearly decreased (0.51-fold) (Figures 7I,J). Taken together, the filtering function of the LN chain is robust despite inflammatory or immune responses and is enhanced *via* adaptive tissue remodeling.

## Discussion

We showed that a specialized lymphatic sinus structure for lymph fluid filtration was constructed in a limited area of LNs, and MSMs localized in this region are suggested to be the chief effectors of the function. The LN chain system in the lymphatic basin acts as a stepwise filter for removing foreign substances and prevents them from leaking into the bloodstream. These findings shed light on previously unrecognized histo-anatomical frameworks and the physiological aspects of LNs within the lymphatic vascular system.

**FIGURE 8:**
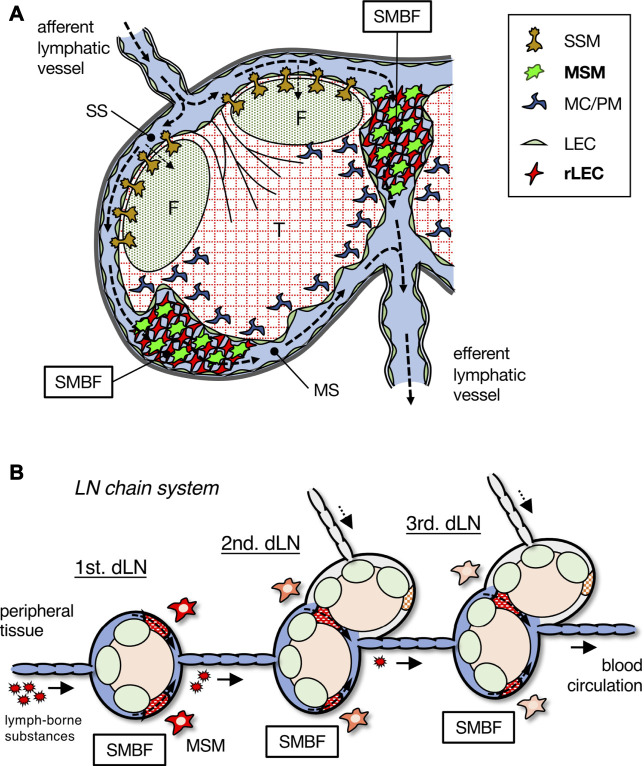
Schematic summary of the SMB filter in LN and the stepwise filtering of the LN chain system. **(A)** The SMB filter (SMBF) is constructed by the network of MSMs and rLECs, which filtrates and removes lymph-borne substances carried by lymphatic flow (dotted arrows). **(B)** The LN chain system in the lymphatic basin. Lymph-borne substances are filtrated by MSMs in the SMB of each LN and gradually removed from lymph fluid. dLN, draining LN.

The filtering function of LNs is confined to the SMB region, in which a unique meshwork structure constructed by rLECs and MSMs has the ability to concentrate and remove soluble or particulate components in the lymph fluid as a physical barrier (Figure 8A). In previous studies, scanning electron microscopy applied to the medullary region of rodent LNs revealed the presence of meshwork-forming cells in the MS lumen, to which several macrophages are attached (Fujita et al., 1972; He, 1985; Ushiki et al., 1995; Ohtani and Ohtani, 2008). Researchers have recognized these sinusoidal cells as LECs and termed them “reticular cells,” “stellate cells,” “retothelial cells,” etc. (Sakuma et al., 1981; Lammermann and Sixt, 2008; Ohtani and Ohtani, 2008). We named these rLECs a critical component of the SMB filter and identified MSMs attached to the reticulum as the effector of the lymph filter. Although the LEC fraction show a small amount of Dex-FITC uptake (<0.5% of the total LN uptake, data not shown), the contribution of LECs is likely to be small in terms of the LN filtering function.

The characteristics of the SMB are consistent with the features of a long-known tissue type, the “reticuloendothelial system,” which is composed of macrophages and sinusoidal endothelial cells (Hoefsmit et al., 1980; Sakuma et al., 1981; Gordon et al., 2014; Zheng et al., 2016). MSMs are known to express a variety of pattern recognition receptors with strong phagocytic activity (Martens et al., 2006; Gordon et al., 2014). For Dex-FITC accumulation, MSMs are assumed to use mannose receptors (MR, CD206) and SIGNR1, which are shown to bind dextran (Gordon et al., 2014; Pustylnikov et al., 2014). MSMs are a macrophage subset similar to splenic marginal zone macrophages, hepatic Kupffer cells, and alveolar macrophages, which share the ability to express MARCO (Van Der Laan et al., 1997; Arredouani et al., 2005; Martens et al., 2006). Interestingly, MARCO is expressed not only in MSMs but also in rLECs in the same region, making MARCO expression the most specific marker to define SMB. A recent study that reported scRNAseq analysis of LECs in murine LNs has also identified a subset defined by MARCO expression, and its distribution is consistent with that of SMB (Xiang et al., 2020).

The significance of the LN filter function being limited to the SMB region is an intriguing issue. The SMB is a narrow area on the lateral part, slightly to the medullary side of the LNs. Although it would not seem inconvenient even if the entire medulla was occupied by filter structures, this is not the case regarding the actual LN, suggesting that it is necessary to concentrate the filtering function in a limited area. As the border of the SS and SMB is the most anterior frontline of the physical barrier, the largest filtration area could be secured by placing it on the lateral part of the organ. In addition, the lymph fluid from the afferent lymphatics entering the LN suddenly changes the flow direction and spreads out to the flat and narrow SS, which would be suitable for SSMs efficiently picking up antigens. Lymph-borne substances captured by SSMs are transported into the follicular parenchyma to induce B cell responses (Szakal et al., 1983; Junt et al., 2007; Phan et al., 2009). In this regard, expanding the SS floor area with the increase of SSMs and setting more follicles beneath seems beneficial for augmenting antibody production. Therefore, this tissue arrangement would be suitable for increasing the efficiency of adaptive immunity.

The SMB filter may also be involved in previously underappreciated responses on the medulla of the LN. We recently found that the deep cortex periphery is a characteristic region in which B cells accumulate in the medullary side of the peripheral T-cell area (Takeuchi et al., 2018). Both B cells and T cells are localized in this part, which directly connects to the intricate MSs and MCs, making them readily accessible to antigens accumulated in the SMB. It is possibly assumed to elicit rapid responses to pathogenic components that quickly reach the MS of LN *via* the afferent lymph.

The chain of multiple LNs in the lymphatic basin plays a crucial role in preventing the spread of foreign substances and leakage into the blood, which is presumed to be particularly effective in inhibiting the spreading of viruses (Figure 8B). By analyzing each LN individually, we succeeded in quantitatively showing the stepwise filtering of lymph-borne substances by the LN chain system. In our experiments, large amounts of tracer were administered for detection, although such doses of infiltration of foreign substances into tissues are unlikely under natural conditions. Therefore, a small number of foreign substances entering the lymph fluid is likely to be mostly trapped by the SMB filter in the first draining LN and rarely reaches the downstream LNs. Even if the most upstream LN misses, the downstream LNs would trap it, providing a safety mechanism for reducing the probability of entering the blood circulation. Moreover, if the filtering function is impaired in the upstream LN for some reason, the filtering efficiency is maintained by a compensatory role of intact downstream LNs.

In mice housed under SPF conditions, the structure of the LNs is relatively simple. However, when inflammatory responses occur, LN tissue is markedly enlarged and remodeled. The mobilization of lymphocytes by inflammation causes the entire LN to expand and dramatically changes the histology, especially in the medulla, leading to enlargement of the sinuses and cord structures (Katakai et al., 2004; Abe et al., 2012; Tan et al., 2012). Although the band-shaped SMB region disappears in hypertrophic LNs, the filtering function of LN is maintained, rather tending to enhance its capacity. Interestingly, an increase in lymph flow into the alternative route, probably owing to the remodeling of lymphatic vessels, augments the uptake of lymph-borne substances in this side pathway. These multiple effects reduce the amounts of foreign substances leaking into the blood. We observed the increase of filtering efficiency in the extensively remodeled LN chain system 1 week after the onset of inflammation, but similar changes may occur in early phases, when it is beneficial to prevent the spread of pathogens immediately after the infection.

In large mammals, including humans, the sizes of the LNs are necessarily large compared with small animals, which allow the lobules to fuse together so that typical tissue architecture, that is, the literal cortex surrounding the medulla, can be formed. In such LNs, the lymphatic sinus in the lobular boundary from the trabecular sinus to the peripheral MS is presumed to correspond to the SMB. However, the histo-anatomical and physiological framework of the lymph filter in human LNs remains unclear. These questions are critical for understanding immune responses in infectious diseases and the development of effective formulations, components, and administration routes in vaccinations. Moreover, the SMB filter in LNs might be closely related to antitumor responses, LN metastasis, and tumor immunotherapies. Further detailed studies are necessary for each situation.

## Data Availability

The original contributions presented in the study are included in the article/Supplementary Material, further inquiries can be directed to the corresponding author.
