# Supplementary material for: Micro- and Macro-Anatomical Frameworks of Lymph Nodes Indispensable for the Lymphatic System Filtering Function
Source: Front Cell Dev Biol. 2022 Jun 20;10:902601. doi: 10.3389/fcell.2022.902601 (PMC9251010; doi:10.3389/fcell.2022.902601)
Supplement: Supplementary file 1 [file DataSheet1.PDF]

Figure S1

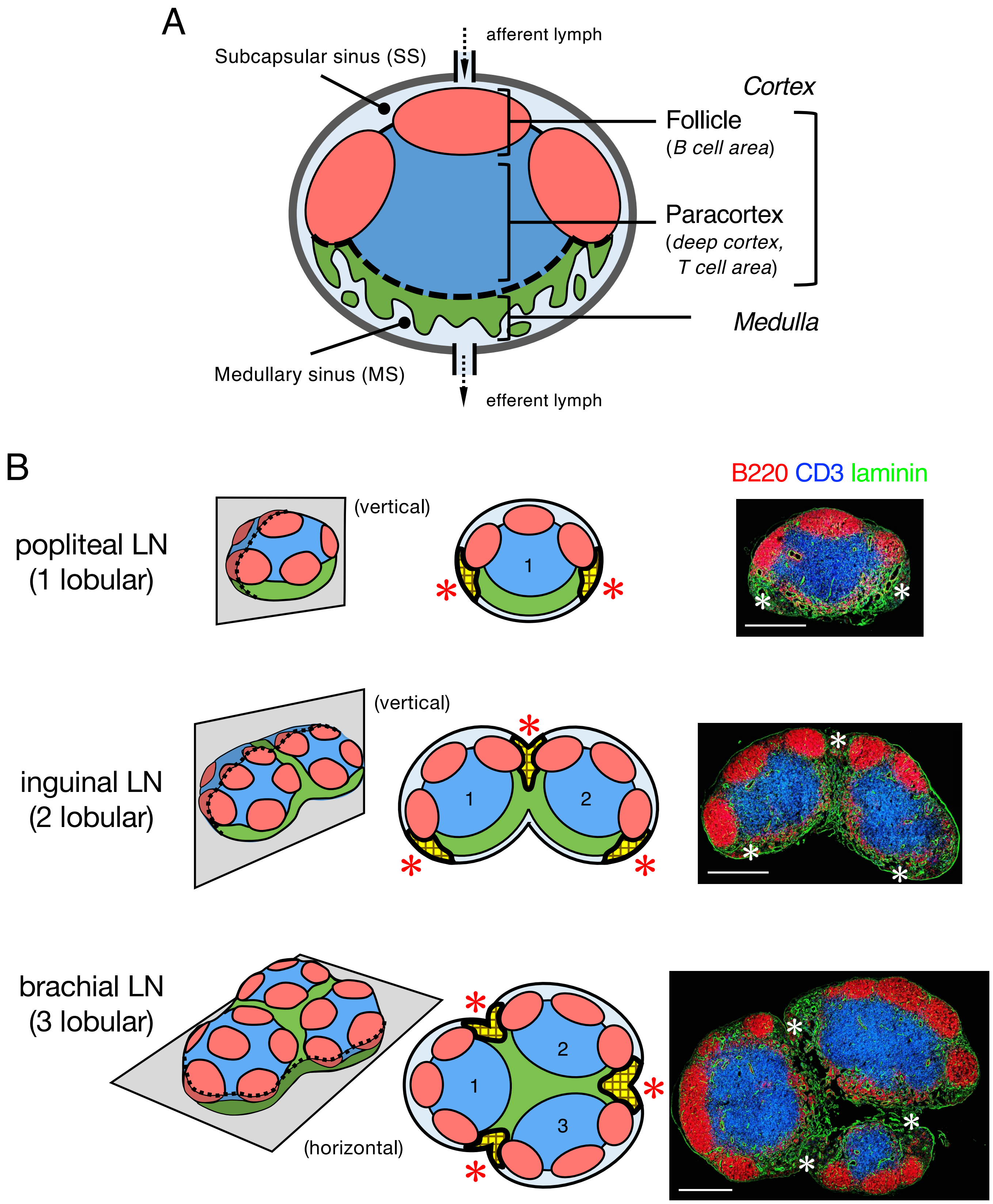

Figure S1. LN structure in mouse.
(**A**) Basic tissue composition of the LN.
(**B**) Lobular compositions in different LNs. Angled views from the cortical side of the LNs (left). The sectional view (middle) of the plane shown in the left and the numbers indicate the lobules in which single T cell area is centered. The transitional region between the SS and MS is highlighted by hatched yellow area (asterisks). Actual confocal immunohistochemical images of LN section correspond to the middle.

Figure S2

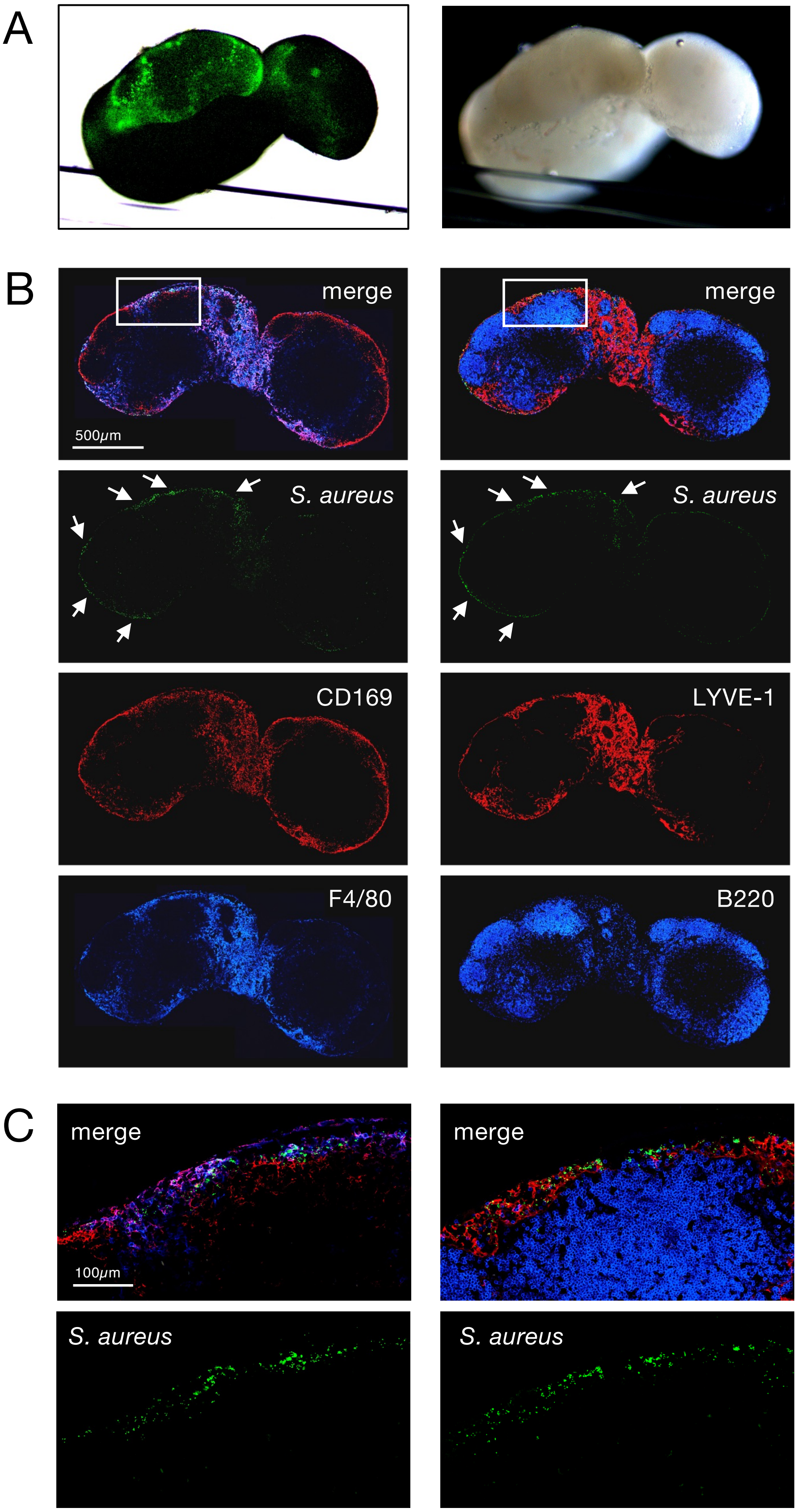

Figure S2. Lymph-borne *S. aureus* mainly accumulates in the SS of LN.
(**A**) Lateral views of the brachial LNs that received fluorescent-labeled *S. aureus*. LNs were excised 1h after subcutaneous injection. Brightfield (right) and transmission/fluorescent composite (left) images are shown.
(**B**) Confocal immunohistochemical examinations LNs that received *S. aureus*. The accumulation of *S. aureus* (arrows) with the indicated antibody staining are shown.
(**C**) Higher magnification views of the SS region in the boxed in (**B**).

Figure S3

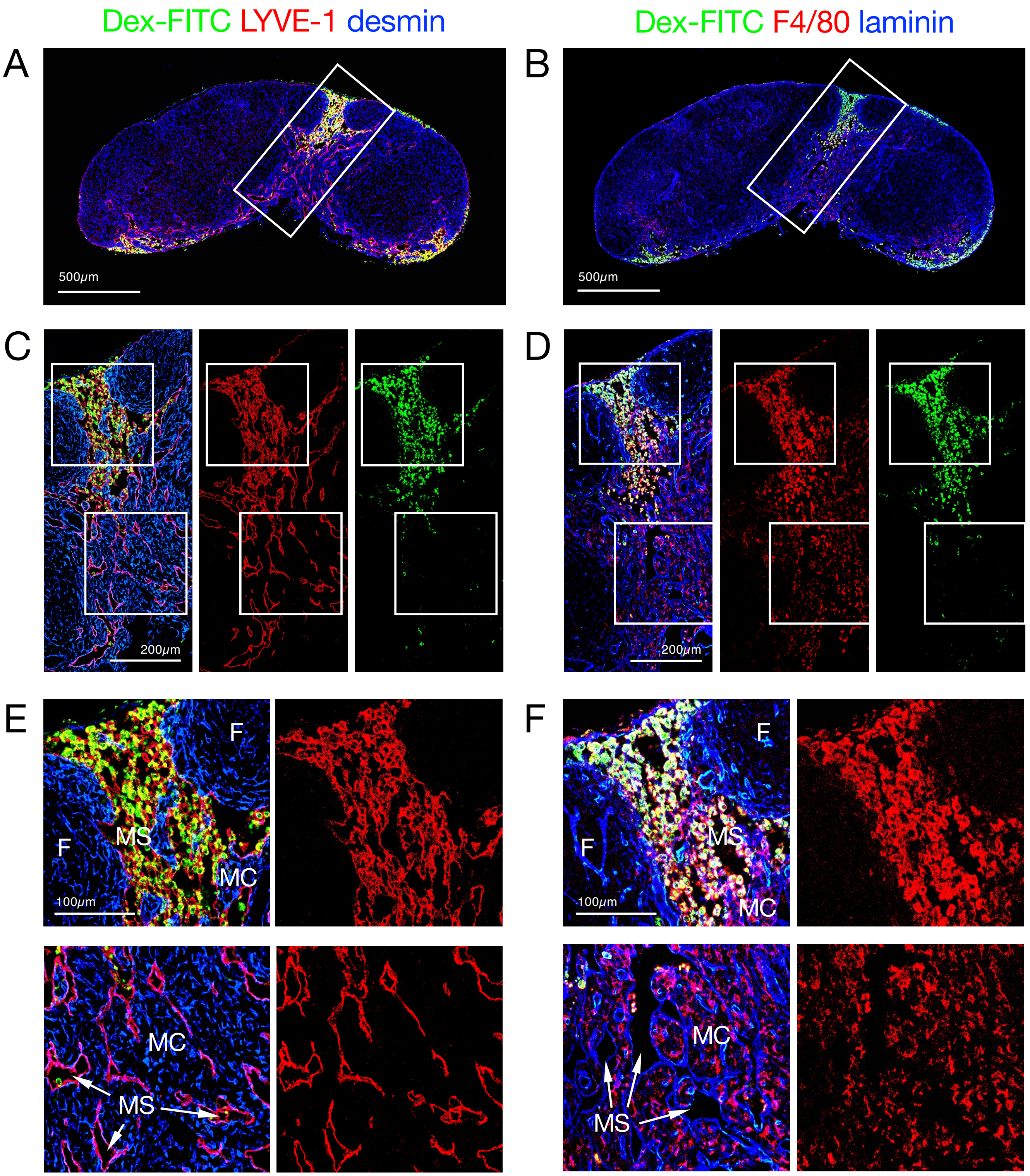

Figure S3. SMB is a specialized region of the MS in which MSMs and rLECs are localized.
(**A, B**) Confocal immunohistochemical images of Dex-FITC–drained inguinal LNs stained with LYVE-1 and desmin (**A**) or F4/80 and laminin (**B**).
(**C, D**) Close-up views of the septal MS between two lobules in the boxed region in (**A**) and (**B**).
(**E, F**) Higher magnification views of the MS subregions; reticular sinus with macrophage accumulation (upper) and simple tubular sinus (lower) in the boxed region in (**C**) and (**D**). F, follicle; MC, medullary cord; MS, medullary sinus.

Figure S4

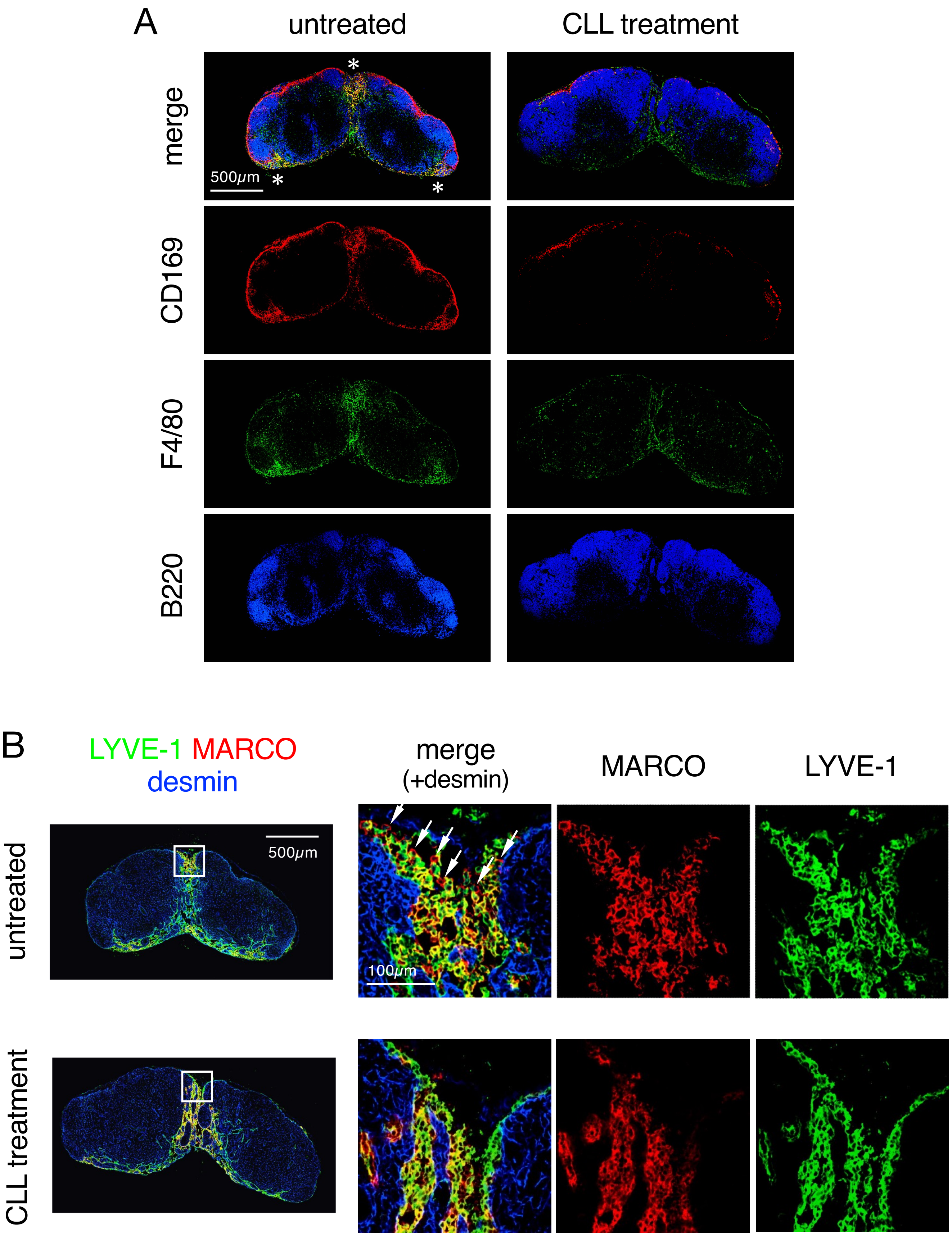

Figure S4. Confocal immunohistochemical examinations of inguinal LN from mice with or without subcutaneous CLL pretreatment. (Related to Figure 3)

(**A**) Asterisks indicate the SMB accumulated with $CD169^{+}F4/80^{+}$ macrophages (yellow) in the untreated control (left), which disappeared in CLL treatment (right).

(**B**) Higher magnification views of the boxed region in the left whole LN image. $MARCO^{+}$ $LYVE\text{-}1^{-}$ cells (MSMs) are found in the untreated control (upper, arrows) but not in CLL treatment (lower).

Figure S5

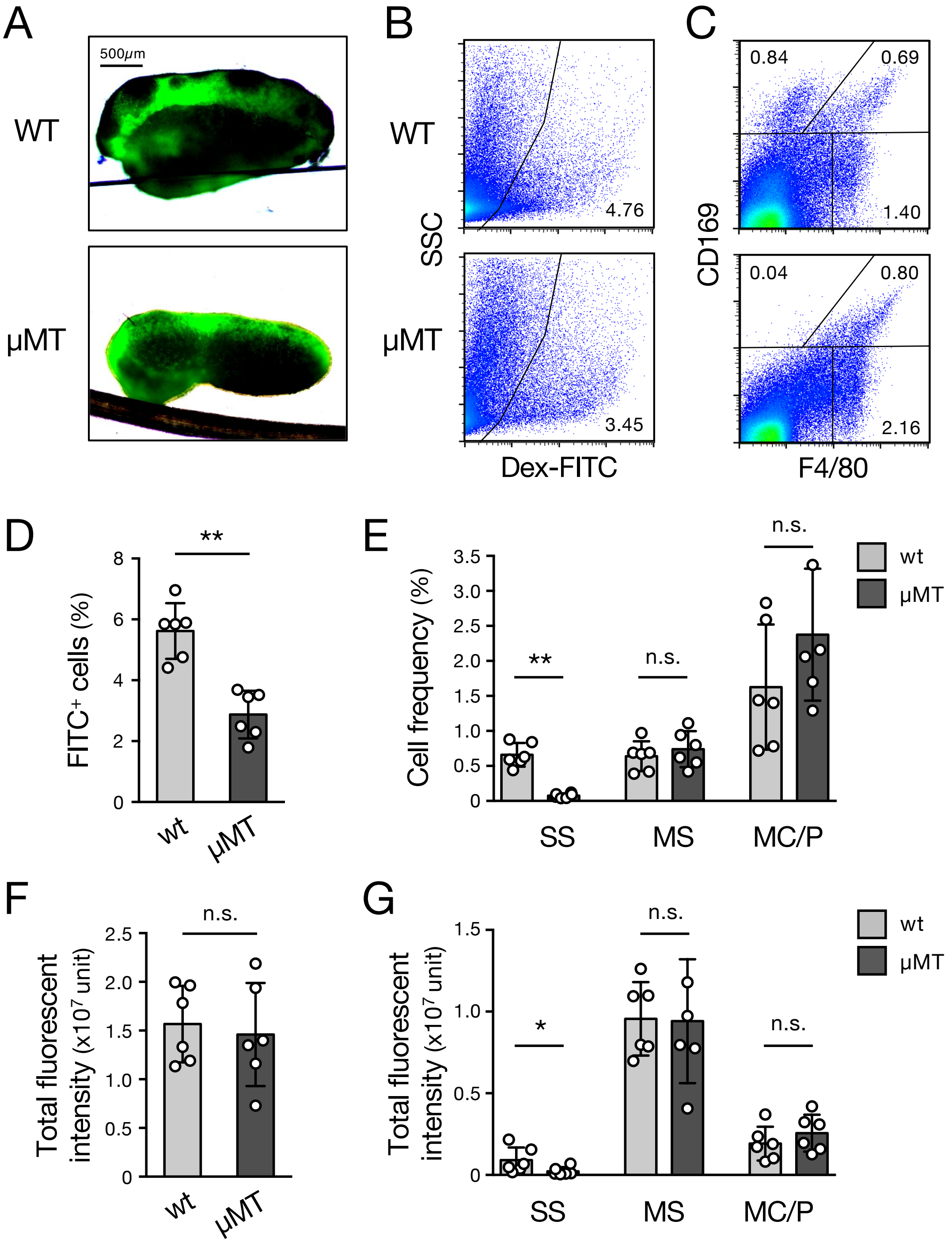

Figure S5. SSMs are dispensable for LN filter function.

(**A**) Transmission/fluorescent composite images of the brachial LN of wt or µMT mice with subcutaneous Dex-FITC injection.

(**B**) Flow cytometric analysis for detecting Dex-FITC^{+} cells in wt or µMT mice LNs. Mice were subcutaneously injected with Dex-FITC (20 µg/site), and brachial, inguinal, and popliteal LNs were excised 1h later for isolating cells by enzymatic digestion. Representative dot plots of flow cytometry are shown. Cells in the right side of the curved line are regarded as Dex-FITC^{+} and the number indicates the percentage in total LN cells.

(**C**) Flow cytometric fractionation of macrophage subsets in LNs by the expression of CD169 and F4/80. Representative dot plots are shown. SSM, subcapsular sinus macrophage; MSM, medullary sinus macrophage; MC/PM, medullary cord/parenchymal macrophage.

(**D**) The percentages of Dex-FITC^{+} cells in total LN cells. Each open circle indicates the result of an individual mouse. Mean ± SD. ** P<0.005. ut, untreated.

(**E**) The percentages of macrophage subsets in LNs. Mean ± SD. ** P<0.005. n.s., not significant.

(**F**) Total fluorescent intensities calculated from mean fluorescent intensity (MFI) and cell number. Mean ± SD. n.s., not significant.

(**G**) Total fluorescent intensities in macrophage subsets. Mean ± SD. * P<0.05. n.s., not significant.

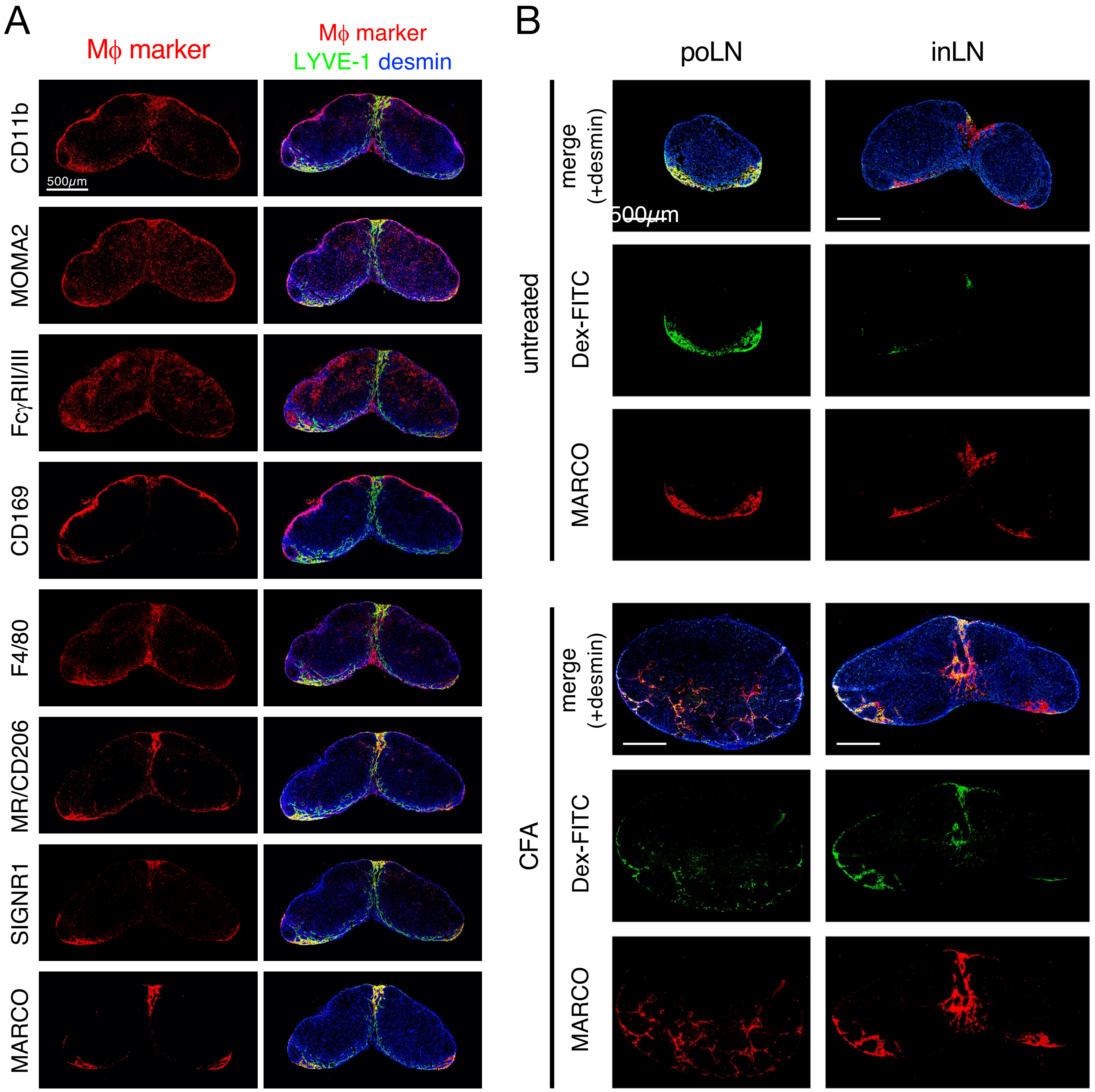

Figure S6. SMB is a unique area characterized by the expression of MARCO.
(**A**) Immunohistochemical screening for identifying the macrophage markers that specifically express in the SMB region of LNs (Related to Figure 4). Serial sections of inguinal LNs were stained for the indicated markers and examined by confocal microscopy.
(**B**) Inflammatory response remodels draining LNs and enhances the filtering efficiency (Related to Figure 7). Popliteal and inguinal LNs from mice pretreated or untreated with OVA/CFA followed by the Dex-FITC injection (0.5 μg). Tissue sections were stained for the indicated markers and examined by SIM fluorescence microscopy.

Figure S7

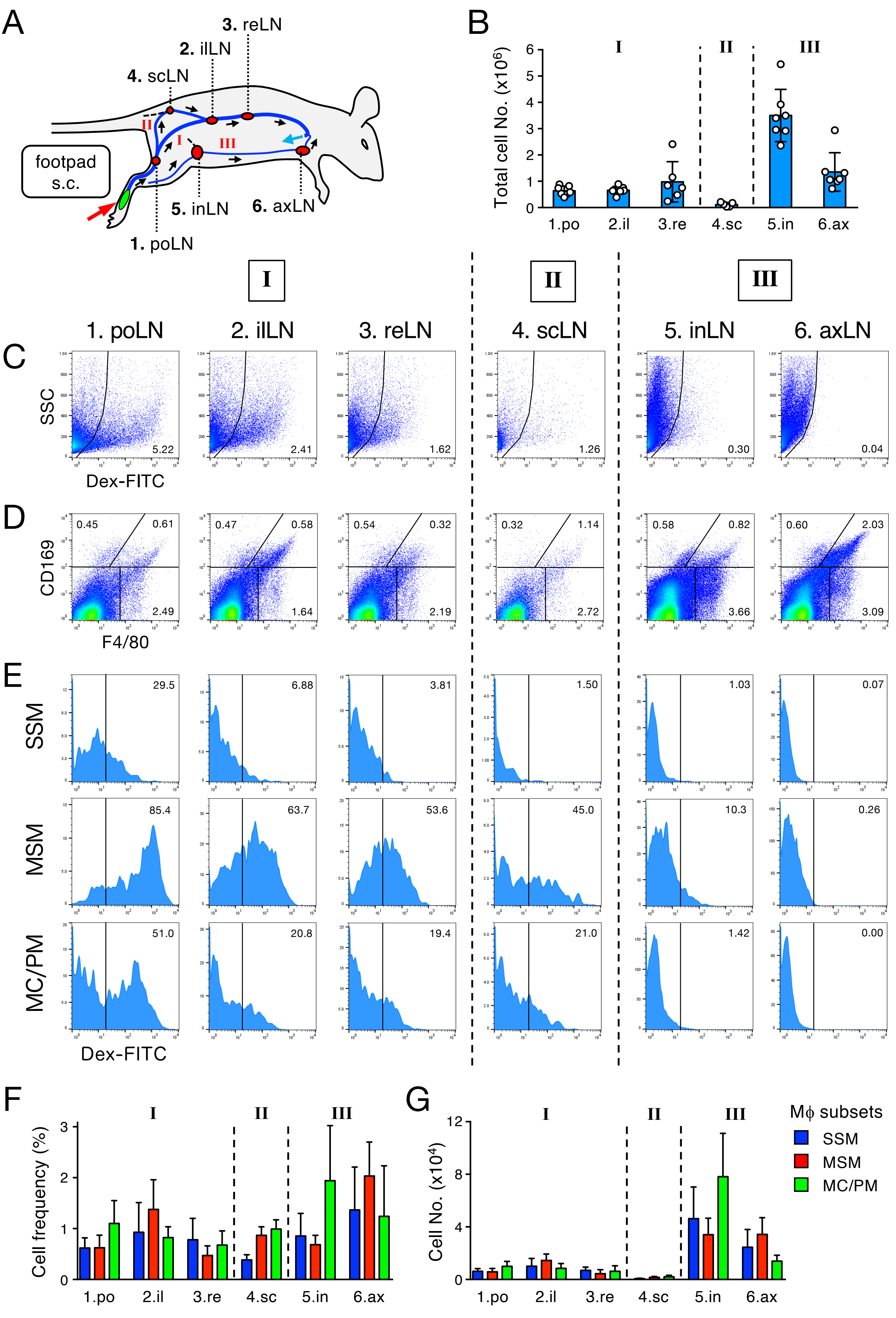
A
footpad s.c.
1. poLN
2. ilLN
3. reLN
4. scLN
5. inLN
6. axLN
I
II
III
B
Total cell No. (x10^6)
1.po
2.il
3.re
4.sc
5.in
6.ax
C
SSC
Dex-FITC
5.22
2.41
1.62
1.26
0.30
0.04
D
CD169
F4/80
0.45
0.61
2.49
0.47
0.58
1.64
0.54
0.32
2.19
0.32
1.14
2.72
0.58
0.82
3.66
0.60
2.03
3.09
E
SSM
MSM
MC/PM
Dex-FITC
29.5
6.88
3.81
1.50
1.03
0.07
85.4
63.7
53.6
45.0
10.3
0.26
51.0
20.8
19.4
21.0
1.42
0.00
F
Cell frequency (%)
G
Cell No. (x10^4)
Mϕ subsets
SSM
MSM
MC/PM

Figure S7. Systematic analysis of individual LN in the lymphatic basin drained from a single site. (Related to Figure 5)

(**A**) Schematic representation of the lymphatic route drained from a single site (hind footpad) of subcutaneous tracer injection and intervening LNs. po, popliteal; il, iliac; re, renal; sc, sciatic; in, inguinal; ax, axillary. The main (I), roundabout (II), and alternative (III) routes.

(**B**) Total cell numbers in individual LN. Each open circle indicates the result of an individual mouse. Mean ± SD.

(**C, D**) Flow cytometric analysis of Dex-FITC^{+} cells (**c**) and macrophage subsets (**d**) in individual LN with subcutaneous Dex-FITC injection (0.5 μg) to the left hind footpad.

(**E**) Histograms for Dex-FITC in each macrophage subset gated in (**d**). SSM, CD169^{+}F4/80^{-}; MMS, CD169^{+}F4/80^{+}; MC/PM, CD169^{-}F4/80^{+}.

(**F, G**) The percentages (**F**) and cell number (**G**) of macrophage subsets in individual LN. Mean ± SD. n=7.

Figure S8

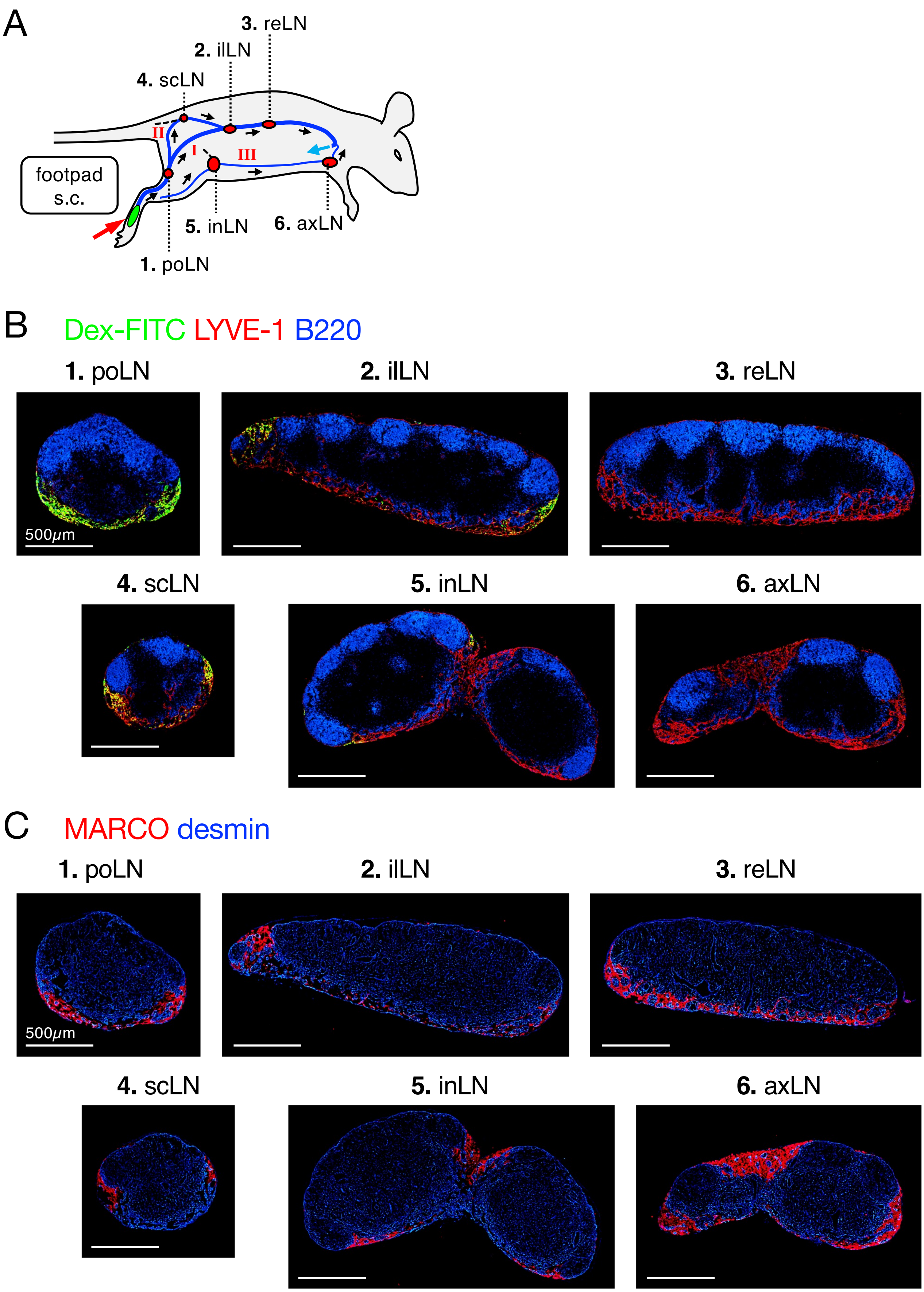

Figure S8. Structural difference of LNs in the lymphatic basin drained from a single site. (Related to Figure 5)
(**A**) Schematic representation of the lymphatic route drained from a single site (hind footpad) of subcutaneous tracer injection and intervening LNs. (Same as Supplementary Figure 6 a)
(**B, C**) LNs from mice injected with Dex-FITC (0.5 μg) into the hind footpad. Tissue sections were stained for the indicated markers and examined by SIM fluorescence microscopy.
